# Regulation of *Staphylococcus aureus* Virulence and Application of Nanotherapeutics to Eradicate *S. aureus* Infection

**DOI:** 10.3390/pharmaceutics15020310

**Published:** 2023-01-17

**Authors:** Kannappan Arunachalam, Poonguzhali Pandurangan, Chunlei Shi, Ricardo Lagoa

**Affiliations:** 1 MOST-USDA Joint Research Center for Food Safety, Department of Food Science and Technology, School of Agriculture and Biology, and State Key Laboratory of Microbial Metabolism, Shanghai Jiao Tong University, Minhang, Shanghai 200240, China; 2 Department of Microbiology, M.R. Government Arts College, Mannargudi, Coimbatore 614001, Tamil Nadu, India; 3 School of Technology and Management, Polytechnic Institute of Leiria, 2411-901 Leiria, Portugal; 4 Applied Molecular Biosciences Unit, NOVA School of Science and Technology, NOVA University of Lisbon, Caparica, 2829-516 Almada, Portugal

**Keywords:** *Staphylococcus aureus*, nanotechnology, drug delivery, antibacterial activity, biofilm inhibition

## Abstract

*Staphylococcus aureus* is a versatile pathogen known to cause hospital- and community-acquired, foodborne, and zoonotic infections. The clinical infections by *S. aureus* cause an increase in morbidity and mortality rates and treatment costs, aggravated by the emergence of drug-resistant strains. As a multi-faceted pathogen, it is imperative to consolidate the knowledge on its pathogenesis, including the mechanisms of virulence regulation, development of antimicrobial resistance, and biofilm formation, to make it amenable to different treatment strategies. Nanomaterials provide a suitable platform to address this challenge, with the potential to control intracellular parasitism and multidrug resistance where conventional therapies show limited efficacy. In a nutshell, the first part of this review focuses on the impact of *S. aureus* on human health and the role of virulence factors and biofilms during pathogenesis. The second part discusses the large diversity of nanoparticles and their applications in controlling *S. aureus* infections, including combination with antibiotics and phytochemicals and the incorporation of antimicrobial coatings for biomaterials. Finally, the limitations and prospects using nanomaterials are highlighted, aiming to foster the development of novel nanotechnology-driven therapies against multidrug-resistant *S. aureus.*

## 1. Introduction

The emergence of drug-resistant pathogens equipped with active defense mechanisms against different classes of antibiotics has become a global threat to human health. Conventional antibiotics belonging to various classes usually act upon bacterial pathogens by interrupting the biosynthesis of genetic materials, such as DNA, RNA and protein, cell wall/membranes, and other cellular components essential for the basic physiology of the bacteria [1]. This traditional approach has been challenged under certain conditions when the pathogens mount resistance mechanism(s) through the expression of antibiotic resistance genes, mutation of drug targets, biofilm formation, and other protective mechanisms. 

The biofilm machinery is a crucial factor contributing to bacterial resistance to conventional antibiotics. Bacterial cells form biofilms by enclosing themselves in or on the surface of a substratum through self-produced exopolysaccharides. The biofilm matrix provides physical and chemical protection to the bacterial population, restraining the attack of antibiotics, host immune processes, and other adverse conditions. Notably, bacterial cells inhabiting biomedical devices as colonies are the leading cause of localized biofilm-associated infections [2].

In this context, *Staphylococcus aureus* is a high-priority pathogen responsible for 80% of hospital infections [3]. In addition, the spreading of drug-resistant pathogens is alarming, requiring novel approaches to combat the emerging drug-resistant strains. Infections attributable to multidrug-resistant (MDR) strains remain a significant cause of mortality and morbidity worldwide. The World Health Organization (WHO) and other health authorities have continuously stressed the concern regarding the threats of MDR strains and their related infections. Furthermore, the lack of new antibiotics in the pipeline worsens the treatment options to combat MDR strains. This alarming situation has prompted research for developing novel and effective antimicrobial drugs, as well as improved drug delivery and targeting methods. Since *S. aureus* is an important pathogen in overcoming antibiotic action, several novel treatment strategies, such as the use of nanoparticles (NPs), are being developed to repress the virulence and subsequent pathogenesis of this superbug. 

Nanotechnology has gained great momentum in recent decades. The research on NPs has attracted much attention for diverse applications, including pollution control, nutraceuticals, medicinal chemistry, biomedicine, and, most importantly, drug delivery [4,5,6,7]. Drug delivery by means of NPs has several advantages over conventional approaches, such as improved drug distribution, controlled drug release, enhanced solubility, bioavailability, and targeting. Owing to its smaller surface area-to-volume ratio, penetration of NPs into biological membranes, cells, and tissues will also be facilitated, which is crucial for drug bioavailability and activity. Three different kinds of NPs are being studied for drug delivery and antimicrobial action, namely organic (polymeric NPs liposomes and others), inorganic/metallic (e.g., silica and metal NPs), and hybrid (combining organic and inorganic components) NPs [8,9]. 

The clinical acceptance of drug-loaded nanoformulations is at an initial stage, and a few products have already been approved for clinical use. For instance, AmBisome^®^ is a liposomal carrier of amphotericin B and can be used against fungal infections [10]. Similarly, Arikace^®^ (amikacin-loaded liposome suspension, aerosol delivery) was approved against *Pseudomonas aeruginosa* in non-tuberculous mycobacterial infections [11].

Considering the importance of *S. aureus* infections and the emergence of drug-resistant strains, several nanotechnology-derived strategies like NPs loaded with drugs and improved antimicrobial surfaces are being developed against this pathogen. Previous reviews focused on the properties and applications of certain nanosize materials for antimicrobial purposes [12,13,14,15,16]. However, several other alternatives are under development, specifically for *S. aureus* pathogenesis. Hence, this review aims to grasp the therapeutic potential of nanotechnology-based approaches combating *S. aureus* infections of clinical relevance.

## 2. Host Defense and Pathogenesis in *Staphylococcus aureus*

### 2.1. Staphylococcus aureus and Its Impact on Human Health

*S. aureus*, a normal flora of skin and mucosa, seizes the opportunity to cause opportunistic infections, most commonly from superficial to invasive infections like skin infections, bacteremia or pneumonia, etc., either as nosocomial or community infections [3]. The evolution of drug-resistant *S. aureus*, especially methicillin-resistant *S. aureus* (MRSA) report in the late 1990s [17] and vancomycin-resistant *S. aureus* (VRSA) in 2002 [18], demanded careful attention as it complicates the treatment. The WHO revealed in 2014 that 86% of clinical *S. aureus* strains were resistant to methicillin [19]. Frequent use of certain antibiotics in the hospital environment has promoted the emergence of MDR strains. Regulation of significant virulence factors and formation of tolerant or persistent subpopulations (persister cells) also adds to the risk of invasive and recurrent infections by *S. aureus* [20]. The cellular expression of surface proteins facilitates bacterial adhesion to the host cell or tissue, thereby causing adverse pathogenesis in humans [21,22]. These proteins are particularly specified to promote bacterial adhesion to the host tissues. Several structural and secreted virulence factors in *S. aureus* play a crucial role during pathogenesis (Table 1).

### 2.2. The Host Immune Response to Staphylococcus aureus

The host immune response elicits the defense machinery against bacterial invasion. Upon bacterial cell invasion, the host immune system instantly brings forth the trapping mechanism by recognizing the antigenic determinant on the pathogen. In addition, the early response signals to other immune cells to localize to the site of invasion or infection. Following pathogen- and host-derived chemotactic factors, host immune cells like neutrophils from the bone marrow or peripheral blood migrate to the site of infection. The host system triggers polymorphonuclear leukocytes (PMNs), which probably stand as the first line of defense. However, there may be slight alterations in PMN response against biofilm-forming *S. aureus* [24,25]. 

Nevertheless, the host pro-inflammatory response at the early stage, which is not considered very effective, leads to the activation of PMNs (i.e., neutrophils/granulocytes) and thereby the release of lactoferrin, elastase, and other relevant components with antimicrobial potential [25]. While the activation of PMNs triggers the localized influx of immune system players at the infection site, it also promotes opsonization with the aid of IgG to eliminate *S. aureus* [24]. In parallel, PMNs also activate phagocytosis to eradicate the free-living planktonic cells by reactive oxygen species (ROS) generation [26], and has a significant role in reducing the biofilms of *S. aureus*. In addition to ROS, the degranulation process (fusion of azurophilic and specific granules with phagosomes) enriches the vacuole lumen of phagosomes containing *S. aureus* with antimicrobial peptides and proteins to ensure bacterial clearance [27]. The azurophilic and other granules comprise enzymes, cytokines, antimicrobial peptides, and other components such as α-defensins, proteinase-3, elastase, cathepsins, azurocidin, lysozyme, myeloperoxidase, etc. [28]. 

The study by Alegre and co-workers documented the immune response in skin and soft tissue infections and hypothesized that their occurrence in healthy persons may be due to a suppressed immune system compared to uninfected persons [29]. On the contrary, the experimental analysis indicated that developing systemic immune responses leads to an elevated level of innate cytokines in infected persons. The same work highlighted the production of interleukins IL-17, IL-22, and IL-10 by macrophages. Further, IL-6 can indicate T lymphocyte differentiation, which promotes Th17 effector or memory cells. Another unique mechanism from neutrophils has been suggested by Brinkmann et al. [30], in which they secrete structures constituting chromatin in a de-condensed form along with histones and cytosolic and azurophilic granule proteins, that effectively act against *S. aureus*. Moreover, staphylococcal toxins stimulate Th1/Th17 responses leading to asthma and allergic rhinitis [31,32].

### 2.3. Immune Evasion by Staphylococcus aureus

*S. aureus* elicits a series of proinflammatory responses to avoid the actions of the host immune system. For instance, protein A and immunoglobulin-binding protein A (Sbi) of *S. aureus* directly bind the Fc portion of IgG to avoid opsonization (antibody-mediated killing) as well as phagocytic activities [33]. Further, *S. aureus* Coa (coagulase) induces the conversion of fibrinogen into fibrin, which assists in protecting the bacterial cell from the host. Peripheral monocytes in the blood promote the release of IL-8, which trigger neutrophil activation. *S. aureus,* however, can induce the synthesis of ClfA (clumping factor A) and SdrE (serine aspartate repeat protein). These proteins prevent the regulatory proteins Factor I and Factor H from activating complement pathways, which cleave the Cd3 domain and block complement pathway-mediated phagocytosis [34,35]. While analyzing the role of complement in *S. aureus* bacteremia, several patients were found with low levels of C3 and C4, pointing to the activation of the classical pathway. However, there were indications of activating the alternative pathway to eliminate the pathogen [36]. In some instances, protein A from *S. aureus* binds to IgA and IgM, preventing complement binding and obviating the classical pathway of complement defense. Hypervirulent *S. aureus* mainly disrupts the overall host defense by influencing antimicrobial peptides, phagocytic elimination, and infection resolution [37]. Likewise, *S. aureus* produces several proteins like the staphylococcal complement inhibitor, chemotaxis inhibitory protein of *Staphylococcus*, extracellular adherence protein, hemolysins, Staphylococcal binding of IgG, aerolysin, phenol-soluble modulins, nuclease, leukocidins, FPR2 inhibitory proteins, and staphylococcal superantigens among others, to evade host immune mechanisms like neutrophil extravasation, priming, chemotaxis, activation of neutrophils, opsonization and phagocytosis, NET formation, and bacterial killing by neutrophils [38].

### 2.4. Challenging Issues of Staphylococcus aureus in Healthcare

The more problematic issues related to *S. aureus* infections are described in more detail below. The outbreak of MRSA has had a vast impact over the years, in a particular assortment from hospital- to later community-based infections, in persons lacking medical attention [20,39]. Nevertheless, in recent decades, MRSA has shown resistance to the extended spectrum of beta-lactam (ESBL) antibiotics, including penicillins, cephalosporins, and carbapenems [40]. However, 80% of the mortality rate in hospitalized infections is due to biofilm formers [3]. The recurrent infection range of MRSA comprises skin and soft tissue infections, bacteremia, endocarditis, urinary tract infection, osteomyelitis, nasal colonization, and cystic fibrosis complications [20]. In addition, MRSA also dominates the list of implant-associated infections [41]. 

The more typical *S. aureus* illness is skin and soft tissue infection, which may include impetigo and purulent cellulitis [21]. Impetigo is common in staphylococcal infections, particularly in the crusty lesion of extremities [29]. Toxic shock syndrome occurs due to toxic shock syndrome toxin [42], primarily caused by the use of absorbable tampons, and it includes serious clinical conditions, such as multi-organ coupled septic shock. Considering the crucial role of *S. aureus* as an inhabitant of nares, Nurjadi et al. [43] underlined the connection between *pvl,* found among intercontinental travelers, and the *lukL*/*lukS* genes associated with the severity of skin and soft tissue infections. MRSA has been reported as a major cause of healthcare-associated pneumonia in the statistical analysis performed by Walter et al. [44] in European countries. Next to *Pseudomonas aeruginosa*, *S. aureus* is a major colonizer of the lungs in patients with cystic fibrosis. *S. aureus* influences cystic fibrosis transmembrane conductance regulator protein present in the epithelia. It leads to mucus accumulation in the respiratory tract, resulting in dyspnea and ultimately to the morbidity and mortality of cystic fibrosis patients [45]. Endotracheal tubes are the primary source of acquiring pneumonia by exhaustive use of ventilators in intensive care unit patients through the development of biofilms [46]. Pacemakers, defibrillators, and heart valve implants are other targets for *S. aureus* cardiovascular infections, which are the more common causes of early-onset prosthetic valve endocarditis [47]. 

Notably, sepsis and endocarditis producing an elevated mortality rate was observed in various vascular catheters [3]. Fibrinogen-binding proteins, such as clumping factors (Clfs) ClfA and ClfB, in addition to SdrE, result in platelet aggregation, which paves the way for endocarditis [48]. Previous research identified *S. aureus* as the second most common species in ventricular shunt infection [49,50]. Intracranial pressure and meningeal irritation were major symptoms described in cerebrospinal fluid shunt infections [51]. An increased risk of shunt infection was observed after the reimplantation of shunts and cerebral spinal fluid leakage in a post-operative crisis. Furthermore, *S. aureus* attempts to cause a viscous infection called osteomyelitis in the case of bones and joints [52]. Another current concern with *S. aureus* is infections during orthopedic procedures, especially knee or hip arthroplasty [53]. 

Urinary tract infections by *S. aureus* are rare. Yet, conditions like older age, hospital exposure, urologic surgical procedures, long-term urinary tract catheterization, urinary tract obstruction, and malignancy favor *S. aureus*-associated hematuria, dysuria, bacteriuria, or bacteremia [54]. The study by Gjodsbol et al. [55] indicated that *S. aureus* is present in more than 80% of chronic wound infections typified by diabetic foot ulcers, venous ulcers, and pressure sores. 

### 2.5. Biofilm Formation in Staphylococcus aureus

A crucial biological tactic that enables bacteria to resist attacks from the host and other threats in a hostile environment is the production of three-dimensional structures termed ‘biofilms’ [56,57]. *S. aureus* engages itself in the formation of biofilms on distinct surfaces and play significant roles in chronic infections in humans [58]. *S. aureus* prevails in biofilm formation on biotic (e.g., host tissue) or abiotic substrata, especially indwelling biomedical devices. To date, *S. aureus* has been reported on various biomedical materials, such as endotracheal tubes, dental prostheses or implants, intravenous catheters, vascular prostheses, intrauterine devices or urinary catheters, cardiac pacemakers, contact lenses, prosthetic joints, and prosthetic heart valves [8]. 

#### 2.5.1. Overview of Biofilm Formation

**Figure 1:**
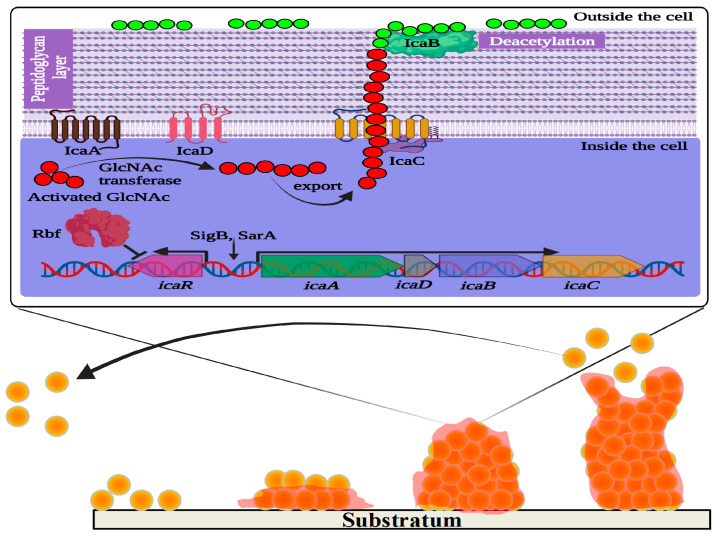
Schematic representation of *ica* operon regulating PIA-dependent biofilm formation in *Staphylococcus aureus*. IcaA and IcaD are two transmembrane proteins involved in the synthesis of N-acetylglucosamine (GlcNAc) oligomers that are 15–20 residues in length from activated precursor UDP-GlcNAc units. The growing GlcNAc chain is likely to be exported by another membrane protein named IcaC, which is also suspected to modify PIA molecules. IcaB is an outer membrane protein that generates positive charges in the GlcNAc polymer molecules by deacetylation. The cationic character of PIA is critical for its role in attachment to the anionic bacterial cell surface. IcaR is a repressor protein that controls the expression of *icaADBC*. Rbf promotes biofilm formation by regulating the expression of *icaR*. In the lower panel of the figure, the four main stages of biofilm formation are depicted: adhesion, colonization, maturation, and dispersion.

To produce biofilms in a biological niche, the population of *S. aureus* follows a sequence of steps: (i) adhesion in a reversible state; (ii) irreversible microcolonization; (iii) propagation of 3D biofilm; (iv) maturation; and (v) dispersion [59,60] (Figure 1). Initial adhesion on the substrate is established by a group of free planktonic bacterial cells. The hydrophobic and van der Waal’s forces facilitate the initial adhesion to the substratum. This non-covalent interaction between the bacterial cells and the substrate is initially reversible. Therefore, the film formed by the early attachment always appears to be fragile. Subsequently, the adhesion becomes irreversible with stronger binding of the bacteria to the substrate. This is made possible as the bacterial population imparts charges on the surface to which it is attached. The conditioning of the film enables the consequent synthesis of specific adhesive proteins and lipopolysaccharides from the *S. aureus* inhabitants. In addition, flagella and pili also play an indispensable role. Quorum sensing is the pioneering machinery followed by the bacterial cells, allowing them to form biofilms via distinct gene expression, and communication among the individual cells of a population (for instance, bacterial cells in biofilm) that relies on signaling molecules described as autoinducers (AI) [59].

#### 2.5.2. Biofilm Matrixome and Dynamics

The stacking of bacterial cells is accomplished by the expression of numerous virulent genes that produce essential components of the biofilm, including nucleic acid (eDNA), proteins, lipids, and polysaccharides that constitute the extracellular polymeric substances (EPS) matrix [60]. As the biofilm matures, the accumulation of components in the EPS matrix consolidates the three-dimensional structure, including its thickness and strength. The multilayered mature biofilm architecture maintains the hierarchy in which the deeper zone encompasses closely packed bacterial cells and persister cells, while the upper zone is more sparsely populated. The persister cells (~1% of the biofilm population) residing in the biofilm tolerate the antibiotics in a dormant or quiescent state, and such action diverges from actual drug resistance [61,62]. The eDNA in biofilm has a vital role in establishing viscoelasticity control, aiding the defensive role of biofilm. 

There may be specific channels for the transport of water and nutrients to carry out a low rate of metabolism. Nonetheless, the cells in the deeper region typically remain dormant, possibly because of insufficient nutrients and oxygen that are depleted at the upper region of the biofilm [63]. Four distinctive states of bacterial cells have been identified in biofilms: aerobic cells, fermentative cells, slow growers/dormant cells, and dead cells [63]. Dead cells are also considered the most crucial source of eDNA that facilitates the live bacterial cells to adhere to the surface [64]. The physiological changes in the cells residing in biofilms (e.g., less motility and hydrophobicity) resist the action of antimicrobials and other stress conditions, like pH, osmotic pressure, chemicals, desiccation, nutrition, temperature, radiation, etc. [60]. Further, the EPS matrix hinders the diffusion/penetration of antibiotics inside the biofilm. Apart from these, active efflux pumps and the divergent expression of certain genes configuring membrane transport have been described as contributing to resistance to various antibiotics [65]. Finally, in the biofilm dispersion stage, cells residing in the biofilm produce proteases and nucleases to degrade the biofilm matrix. Once the biofilm matrix degrades, the cells leave the biofilm and enter the planktonic stage again to initiate the adhesion on another surface or continue their life cycle as free planktonic cells to infect other body parts [58]. The biofilm dispersion may be mechanical, through a fluid run of the host, or genetically programmed [66]. The following section discusses distinct genes involved in biofilm formation and their contribution in *S. aureus* virulence.

Bacterial cells undergo unique gene transfer mechanisms within the biofilm through conjugation, transformation, and transduction [67]. With horizontal gene transfer mechanisms, the resistance gene(s) prevailing in a single bacterium can be transferred to other cells. Thus, biofilm formation enables the development of antibiotic-resistant strains through the regulated production of the EPS matrix and the horizontal transfer of antibiotic-resistance genes. 

### 2.6. Virulence Regulation in Staphylococcus aureus

#### 2.6.1. Regulation of the Two-Component System AgrAC in *Staphylococcus aureus*

Heterogeneous gene expression has been found in *S. aureus* to regulate its virulence in most conditions. Previous studies assessed gene regulation contributing to the virulence pattern in *S. aureus*, for example, those that are induced by low levels of antibiotics [68]. Production of virulence factors and biofilm formation in *S. aureus* is responsible for causing a wide range of infections. Many of them are controlled by the quorum sensing (QS) system in *S. aureus,* named the accessory gene regulator (*agr*) system. QS allows gene expression that regulates the physiological activity of an individual/population, promoting pathogenesis and antibiotic resistance.

**Figure 2:**
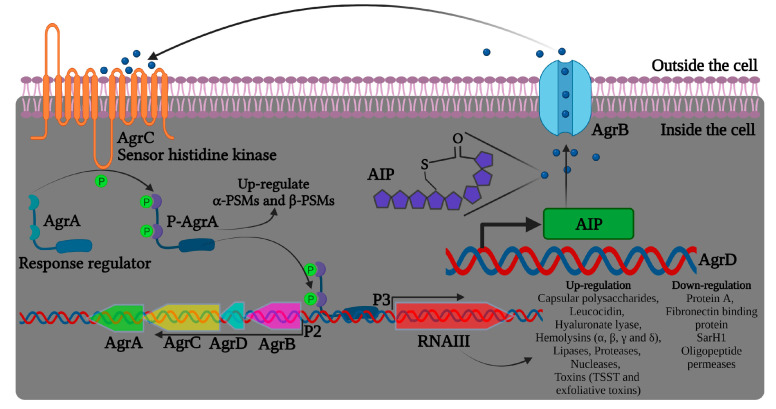
Illustration of the *agr* quorum sensing system in *Staphylococcus aureus* and RNAIII regulation of virulence factors. The precursor AIP is encoded by the *agrD* gene. AgrB is a transmembrane protein likely to be involved in the maturation and transport of the precursor AIP. AIP can bind to AgrC that, together with AgrA, form a TCS signaling system and increase *agr* activity.

Bronesky et al. (2016) have described that the induction of virulence factors in *S. aureus* is regulated by the complex of genes in an *agrACDB* cassette possessing two signaling transducing modules, a receptor histidine kinase (HK) *agrC*, and a cytoplasmic response regulator (RR) *agrA*, as represented in Figure 2 [69]. The *agrC*-*agrA* modules form a classical two-component system (TCS) composed of an HK sensor and a RR protein, important in bacterial responses to environmental factors, such as antibiotics and QS signals [70]. The locus *agrD* encodes the precursor of autoinducing peptide (AIP), and AgrB functions as an endopeptidase and chaperone protein involved in the maturation and release of active AIP. The *agr* system gets activated upon reaching the threshold level of AIPs in the external environment. Upon binding with AIPs, AgrC phosphorylates AgrA, which binds to the region between the *agr* promoters, activating both P2 and P3 for the synthesis of RNAII (*agrA-D*) and RNAIII transcripts, respectively (Figure 2). The transcription factor *agrC* thus completes a strong positive feedback loop and upregulates the expression of α-phenol-soluble modulin (PSM) and β-PSM proteins. The hairloops of RNAIII upregulate or suppress target genes by offering transcriptional/translation control. For instance, RNAIII binds to *hla* ribosomal binding site and promotes α-hemolysin (*hla*) translation in *S. aureus* [71]. Similarly, RNAIII differentially regulates the expression of several virulent genes, including hemolysins, repressor of toxins (Rot), toxic shock syndrome toxins (TSST), and several others, as listed in Figure 2. 

Apart from the *agr* system, there are other TCS with important roles associated to *S. aureus* virulence (Table 2) and are considered appealing targets for developing antibacterial drugs [70].

In addition to the autoregulatory nature of AgrA, P2 and P3 promoters can be regulated by other players, such as SarA/SarR (staphylococcal accessory regulator), with SarA able to activate and SarR repressing P2 transcription [72,73]. It was also reported that the TCS *srrAB* and the global regulator CodY manipulate the *agr* system expression [74]. Moreover, the *agr* QS system was described to mediate biofilm dispersion by upregulating the extracellular protease in conditions of appreciable biofilm population and low nutritional level [75,76]. 

**Table 2:** Summary of the role of two-component systems and other regulatory genes involved in the virulence genes expression in *Staphylococcus aureus* [77,78,79,80,81,82,83].

Regulators	Functions
AgrCA	Cell-to-cell communication system, where the bacteria communicate with self-produced autoinducing peptide Essential for biofilm disassembly and initial attachment Agr regulation represses adhesins and stimulates phenol-soluble modulins and proteases
AirSR/YhcSR	Involved in cellular homeostasis and energy production Important for aerobic and anaerobic growth Positively regulates the expression of the *sspABC* operon
ArlRS	Positive regulator of MgrA and Spx Regulates many cellular processes, including cell wall-anchored adhesins, virulence factors, polysaccharides, and capsular synthesis genes
BceRS	Positive regulator of *bceAB* and *vraDE* genes Confers resistance towards bacteriocins by transporting bacteriocins outside the cytoplasm through BceAB and VraDE proteins
BraSR	Confers resistance towards nisin A and nukacin ISK-1 Exhibits significant regulatory effects on the symbiosis of *S. aureus* and the type I bacteriocin strain
CodY	Strain-dependent regulation of PIA Positive regulator of biofilm formation through the induction of *ica* operon Cytoplasmic regulator for metabolic response Positive regulator of virulence factor protease
GraRS/ApsRS	Belongs to the intramembrane-sensing histidine kinase (IM-HK) familyPositively regulates expression of the *dlt* operon Essential in evading host defense mechanisms such as neutrophil killing and cationic AMPs
HptRS	Hexose phosphate transporter Primarily involved with hptA (initiates autophosphorylation of *hptS* based on the phosphate concentration), *uhpT* (downstream regulatory protein transports phosphate/fosfomycin into the bacterial cell to maintain physiological metabolism) Mutation reduces the uptake of fosfomycin (structure similar to phosphoenolpyruvate) and increases the bacterial resistance
HssRS	Heme sensor system responding to heme exposure Positive regulator of the efflux pump HrtAB (heme-regulated transporter), which plays a role in maintaining intracellular heme homeostasis Found to have a role in regulating virulence and modulating host immune response
KdpDE	Involved in sensing potassium (K+) limitation or salt stress It plays a role in the expression of genes involved in capsule biosynthesis, amino acid and central metabolism
LytRS	Regulates cell lysis and induces the expression of *irgAB* Plays a predominant role in eDNA-mediated biofilm formation
MgrA	SarA family cytoplasmic regulator and prime effector of the ArlRS system Repression of adhesins and negative regulator of biofilm formation
NreCB	Involved in oxygen sensing; converts nitrate and nitrite as final oxygen acceptors Regulates the gene clusters involved in nitrate (*narGHJI*) and nitrite reduction (*nirRBD*) Expression is controlled by NreA, which inhibits NreB autophosphorylation
NsaRS	Belongs to the IM-HK family Important in conferring resistance towards nisin
PhoRP	Involved in response to inorganic phosphate starvation Positive regulator of *pstSCAB* and *nptA*, and also modulates the expression of *pitA*
Rot	SarA family cytoplasmic regulator and prime effector of the Agr QS system Positive regulator of biofilm formation through protease repression and adhesin induction
SaeRS	Stimulates adhesin and nuclease production and is very much crucial in the biofilm maturation process
SarA	Positive regulator of biofilm formation through the induction of *ica* operon Induces biofilm formation through the repression of protease production
SigB	Strain-dependent regulation of PIA Positive regulator of virulence factors protease and nucleaseEssential for initial attachment and biofilm disassembly Cytoplasmic regulator for stress response
SrrAB	Global regulator for *S. aureus* virulence Critical for survival under environmental stress Regulation of genes involved in anaerobic metabolism, nitrous oxide detoxification, cytochrome biosynthesis and assembly, biofilm formation, hydrogen peroxide metabolism, and programmed cell death
VraRS	Associated with vancomycin resistance Essential for cell wall synthesis Positive regulator of PBP2, SgtB, and MurZ
WalKR/YycGF	Essential for cell wall metabolism and cell viability Positive regulator of AtlA, SsaA, IsaA, and LytM Regulates the expression of host matrix interaction proteins, cytolytic toxins, and proteins involved in host immune evasion

#### 2.6.2. Intercellular Adhesin-Mediated Biofilm Formation and Beyond

Although several factors were reported to influence the attachment and subsequent biofilm formation, the production of extracellular polysaccharide adhesin termed polysaccharide intercellular adhesin (PIA) or polymeric-N-acetylglucosamine (polyGlcNAc) is the best studied biofilm mechanism in Staphylococci [84]. PIA is produced by proteins encoded by the intercellular adhesin (*ica)* gene cassette, including *icaR* and *icaADBC* (both regulatory and biosynthesis domains). During biofilm formation, the regulator of biofilm formation, Rbf protein, facilitates the expression of the *icaADBC* operon by suppressing *icaR* expression [85]. In addition, the *ica* gene cassette is essential for adhesion to surfaces, like medical devices, by mediating the deacetylation of PIA to produce more positively charged polyGlcNAc molecules [86] (Figure 1). However, biofilm development can also be enhanced in an alternative mode (PIA-independent synthesis), even in the absence of the *icaADBC* operon, especially in *ica* mutant *S. aureus* [84]. In such *ica* mutant strains of *S. aureus*, biofilm production is promoted by *Staphylococcus* surface protein (SasG) and other biofilm-related proteins, like SasC and protein A (SpA) or accumulation-associated protein or biofilm-associated protein. The extracellular matrix-binding protein (Embp) is another significant player underlying Staphylococcal biofilm formation [87]. SasG is a member of the G5-E repeat family, containing G5-E domains and A domain. The N-terminal region of the A domain is linked to cell wall-spanning and anchoring domains. Sequential cleavage of the A domain leads to the exposure of G5-E repeats in the cell membrane, which promote biofilm formation by the adhesion of the G5-E domain of neighboring cells [87,88]. 

LuxS is another notable QS system in *S. aureus* and also in other Gram-positive and Gram-negative bacteria. It has been reported that the autoinducer-2 (AI-2) molecule produced by the *luxS* gene *in S. aureus* controls the transcription of the *ica* operon by repressing the expression of the *icaR* gene [89]. In addition to the PIA synthesis, cellular aggregation in *S. aureus* has also been mediated by FnBPs (fibronectin-binding proteins) with the aid of Atl (autolysin) and *sigB* regulation [90]. SigB is a positive regulator of biofilm formation by enhancing the synthesis of Clf, FnbpA, and coagulase and repressing the proteins involved in biofilm dispersion [91].

### 2.7. Role of Mobile Genetic Elements in Antibiotic Resistance and Pathogenesis in Staphylococcus aureus 

Mobile genetic elements (MGE) or transposons are responsible for carrying the genes that facilitate resistance to antibiotics, such as methicillin or other β-lactams, in MRSA. The staphylococcal chromosome cassette *mec* (*SCCmec*) is one such MGE in *S. aureus* [92]. The *SCCmec* gene cassette comprises a functional domain (*mecA*, methicillin resistance determinant), regulatory domain (*mecR1* and *mecI* representing promoter and repressor, respectively), insertion sequence, and *ccr* (cassette chromosome recombinase gene complex) domain. The *ccr* domain is responsible for mobilizing the transposon (while integrating into *S. aureus* DNA) using its recombinase activity. With the att (attachment) site, the integration of the MGE with the bacterial genome is established at a specific site near the origin of replication, *OrfX* [93]. Malachowa and DeLeo [94] have sorted out several types of MGE in *S. aureus* via plasmids, transposons, lysogenic phages, pathogenicity islands (SaPI), and the staphylococcal cassette chromosome (SCC). It was reported that the antibiotic resistance element *SCCmec* also carries a cytolysin gene encoding PSM. The transfer of such MGE with antibiotic and virulence determinants confers increased virulence, even in the strains that lack genome-encoded PSM production [95]. Similarly, the presence of VanA in plasmid confers resistance towards vancomycin in *S. aureus*, and is believed to be acquired from transposon Tn1546, obtained initially from vancomycin-resistant *Enterococcus* [96]. 

The genetic analysis of *S. aureus* variant strains from a broadly infected population showed a correlation containing similar and related markers, such as the *SCCmec* domain and the Panton-Valentine leukocidin (*pvl*) locus [37,97]. This investigation established the relevance of the *pvl* gene in the formation of abscesses and furuncles [37]. The virulence of MRSA associated with community infections is owing to the expression of certain factors like PVL toxin, alpha toxin, and PSM protein (responsible for the degradation of host neutrophils and WBCs) [98]. PVL is known as a pore-forming protein in the leucocyte membrane, leading to tissue necrosis [99]. Nevertheless, the expression of toxins can be regulated by the *agr* operon and other regulatory genes [100]. 

Given the importance of the difficulties raised by the virulence factors mentioned above and the emergence of multidrug-resistant *S. aureus* strains, the tactics for control and eradication have become progressively sophisticated. Herein, we discuss the therapeutic potential and recent updates of nanotechnology-based treatment paradigms to control the pathogenesis and subsequent infection of *S. aureus*.

## 3. Nanotechnology-Based Approaches to Target *Staphylococcus aureus* Pathogenesis

Nanotechnology offers outstanding alternatives to circumvent the drawbacks of conventional therapeutics. In microbiology, nanotechnology provides effective solutions to combat biofilms and their related infections by employing drug-loaded NPs [101]. The nano-sized particles exhibit a relatively greater surface area, representing an advantage to penetrate the cell membrane and undermine the viability of bacterial pathogens [102]. Indeed, knowledge regarding nanomaterials’ characteristics, mechanism, and transport are essential criteria for designing appropriate NPs with antimicrobial potency [103]. 

In general, NPs can be classified into inorganic and organic NPs. Inorganic NPs include metal-, metal oxide-, and carbon-based NPs, such as carbon nanodots (CNDs). Organic NPs include liposomes, polymer NPs, lipid- and protein-based NPs, dendrimers, and micelles. Drug-loaded NPs present appealing features, as they increase drug stability and bioavailability and can reduce toxicity. Most importantly, drug-loaded NPs aid site-specific delivery of drugs and enhance the penetration and transport of the drugs to improve their efficacy [104,105]. Thus, the use of NPs is envisaged as an alternative treatment regime to inhibit the growth and biofilm development of MDR organisms, especially against *S. aureus* and its associated infections. Table 3 and Table 4 present recent and insightful studies on the ability of NPs to inhibit *S. aureus* growth, biofilm formation, and virulence factors. Mostly published in the last two years, the works cover a diversity of NP types, preparation methods, and applications. In the following sections, nanoformulations characterized with anti-staphylococcal activity will be discussed. Applications of antibacterial nanostructured coatings and insights into the synergy of nanomaterials and antibiotics will also be presented.

### 3.1. Synthesis of NPs

The choice of an appropriate synthesis method is critical for producing NPs in an application-oriented manner [106]. Several methods have been developed over time to synthesize NPs with desired features and have been categorized according to two strategies, namely, bottom-up and top-down strategies [107]. A bottom-up method is a simple approach where the building blocks are added to each other to synthesize the nanostructures. In this method, atoms, molecules, and other chemicals below nano sizes can be utilized as the building blocks for forming the required NPs. Bottom-up NPs have significant benefits in terms of cost, uniformity, and scalability, and also provide more possibilities to synthesize NPs with minor defects, improved size and shape characteristics, and homogenous chemical composition. Synthesizing NPs using a bottom-up strategy includes chemical reduction, photochemical, sonochemical, and biological methods [108,109,110].

The top-down approach is a simple method that involves either the removal or division of bulk materials into nanoparticulates while maintaining the integrity of the original material. The standard techniques employed in top-down strategies are wire explosion, arc discharge, electron beam, laser ablation, and others [111,112,113,114]. However, using top-down approaches has certain limitations related to crystallographic impairment to the processed pattern and surface imperfections, which will create subsequent difficulties in applying and fabricating NPs. This top-down approach is much more suitable for nanofabrication and requires extensive and expensive instruments [107].

### 3.2. Inorganic NPs 

Noble (gold, silver, and platinum) and other metals (zinc, copper, iron, titanium, aluminum, and magnesium) are known to have antibacterial and antibiofilm properties against a wide range of MDR pathogens without a significant risk of resistance development (Table 3). Moreover, metallic NPs can deliver drugs at targeted sites without immune activation and with very low toxicity. Comparable to metallic nanocarriers, mesoporous silica NPs (MSNs) have been extensively investigated as drug carriers because of their stable structure, functionalizable surface chemistry, biocompatibility, and safety [115]. Due to its applicability in disease diagnosis and treatment, the research on metal NPs is at the pinnacle [116]. 

#### 3.2.1. Gold NPs

Gold is considered more inert than other metals and exhibits reduced toxic effects because of its low reactivity. Generally, gold can be found in oxidized states such as aurous (Au^+^) or auric (Au^3+^), or the non-oxidized state (Au^0^) with increased stability that is desirable for gold NPs (GNPs). In the process of GNPs synthesis, reduction and stabilization are the two critical steps to deal with. In the reduction process, the reducing agents act as electron donors and reduce the gold from an oxidized to a non-oxidized state. Stabilizing agents prevent aggregation by providing repulsive forces to the synthesized NPs [117]. 

GNPs have garnered much attention because of their attractive biomedical applications. GNPs have been studied as an effective means for treating various human ailments, including infectious diseases caused by *S. aureus*. Furthermore, the non-toxic and non-immunogenic natures of GNPs with high membrane permeability and increased retention time, highlight the use of GNPs as an antibacterial agent. GNPs may be synthesized via traditional chemical or green synthesis methods using extracts from bacteria, fungi, and plants [118]. Several studies have put forward the use of GNPs as antibacterial agent, but some controversies still exist [119,120]. Notably, the ultra-small GNPs with a particle size of 0.8 nm showed better anti-staphylococcal activity than the 1.4 nm GNPs, suggesting the size-dependent activity of the GNPs [121]. Further comparing their activity, GNPs were more effective in eradicating the Gram-positive Staphylococci and their biofilms than the Gram-negative bacterial pathogens. The GNPs showed acute toxicity to planktonic cells and reduced the viable bacteria by 5 logs after an exposure time of 5 h. Gouyau et al. [122] synthesized citrate-capped GNPs via the Turkevich chemical method with a uniform particle size of 12 nm, which showed a potent staphylococcal inhibitory activity. Although the GNPs have biomedical applications, the chemical synthesis of GNPs has an issue with the presence of impurities like organic solvents, toxic compounds, and other unsafe by-products [123]. To surpass these drawbacks, the synthesis of NPs requires simple and eco-friendly approaches. The green synthesis or biological synthesis of NPs utilizes natural compounds or extracts from bacteria, fungi, or plants, that act as reducing and stabilizing agents [116]. In green synthesis, chemicals or extracts from plants or microorganisms are added to a metal solution to synthesize NPs. Besides antibacterial activity, several reports have demonstrated the antibiofilm activity and persister inhibitory potential of GNPs against *S. aureus*. For instance, Khan et al. [124] synthesized caffeine-loaded GNPs using a green chemistry approach and studied their antibacterial capacities. These particles (Table 3) showed potent inhibition of biofilm formation by different bacteria and activity against stationary phase cells and antibiotic-induced and biofilm-associated persister cells of *S. aureus*. In another study, using *Piper betle* extract as the stabilizing and reducing agent, Bar (2021) synthesized GNPs through a one-pot synthesis method with an average size of 30–50 nm and studied the antibacterial activity of GNPs against four bacterial pathogens, including *S. aureus* [125]. 

In general, NPs exhibit appealing features like the possibility to alter surface characteristics and stability. Meanwhile, the size and shape of the NPs also determine their antibacterial potential. Hameed et al. [126] recently studied the anti-staphylococcal activity of GNPs with different shapes, such as nanospheres, nanostars, and nanocubes. Among the structures studied, nanocubes displayed potent antibacterial activity against *S. aureus* growth. Similarly, Penders et al. [127] also studied the shape- and size-dependent activity of GNPs against *S. aureus*. Nanosphere GNPs and nanoflower- and nanostar-shaped GNPs showed good antibacterial activity and no sign of toxicity towards human dermal fibroblast cells. The results obtained from the above studies highlight the importance of shape and size to the activity of GNPs as an antibacterial therapeutic agent.

#### 3.2.2. Silver NPs

Owing to outstanding results, the application of silver NPs (SNPs) is extending to diverse fields, from medicine to domestic uses. Synthesis of SNPs can be attained via different methods like sol-gel, hydrothermal, biogenic, chemical-vapor deposition, and others, where the Ag^+^ ions are converted to Ag^0^ by various electron donors [128,129]. 

SNPs have been extensively studied as antibacterial agents against free-floating cells and as antibiofilm agents against a broad range of microbial pathogens such as bacteria, fungi, and viruses. Although the mechanism of bacterial inhibition has not been entirely elucidated, some studies point to possible actions of SNP against MDR pathogens, which include the binding and accumulation of positively charged SNPs to the negatively charged bacterial cell membrane, SNPs cell penetration, interaction with proteins and nucleic acids, and the ROS production [129]. More importantly, the SNPs can be efficiently used against MDR pathogens, as the emergence of drug-resistant strains is very low because of its unique and multimodal inhibitory mechanism [130]. Moreover, the biogenic synthesis of SNPs recently gained interest because of the simple one-step process that did not produce toxic chemicals. To circumvent the use of harmful substances, numerous works have described the synthesis of antimicrobial SNPs using natural resources [128,131]. Biogenic SNPs were described to target redox mechanisms and efflux pumps in *S. aureus* (Table 3). In a work combining varied techniques, Goswami and co-workers demonstrated the antibiofilm activity of SNPs synthesized using tea extract against *S. aureus* grown in silicone tubes and polystyrene coverslips [110]. Moreover, the tea extract-produced SNPs were assessed for their toxicity using a hemolytic assay with goat erythrocytes as the substrate. The assay indicated that the hemolytic activity was insignificant and the SNPs were suggested for potential use in formulations to prevent biofilm-related infections. However, cytotoxicity studies with animal cell lines or in vivo animal models to assess SNPs biocompatibility are needed [110]. 

Apart from the role of antibiotic resistance and biofilm formation in limiting treatment options, the intracellular presence of pathogens, especially *S. aureus*, is another upcoming issue responsible for persistent infections. Although *S. aureus* was known to be an extracellular pathogen, recent studies have evidenced the ability of this pathogen to survive intracellularly [132,133]. Addressing this issue, Kang and co-workers showed the potential of SNPs to effectively eradicate intracellular and extracellular *S. aureus* compared to conventional antibiotics like gentamycin, rifampicin, and vancomycin [133]. In this work, smaller particles were more efficacious (40 vs. 100 nm), and higher concentrations were necessary to attack the intracellular pathogen compared to the extracellular counterpart, but with low toxicity towards osteoblast cells.

#### 3.2.3. Copper NPs 

Copper NPs (CuNPs) can also be synthesized either by green or chemical synthesis, in both cases by reducing Cu ions [134]. CuNPs are active against MDR pathogens and impede their biofilms. There are multiple ways through which CuNPs achieve antibacterial activity against MDR pathogens. At first, copper interacts with the thiol groups of key enzymes and proteins, affecting their metabolic activity. Second, reducing cuprous oxide from Cu^+^ ions leads to the formation of peptides with the cell membrane. Finally, Cu^2+^ dissociation from cupric acid provokes ROS, interfering with nucleic acid synthesis and other metabolic and biochemical processes like electron transfer, nitrogen metabolism, and active transport [134]. CuNPs as oxidant agents are sensitive to air, so the synthesis requires non-aqueous media and inert gases to avoid the formation of Cu oxides with reduced antibacterial activity. However, capping CuNPs can prevent the formation of CuONPs [134]. 

A recent study with CuNPs prepared using the medicinal plants *Zingiber officinalis* and *Curcuma longa* (Table 3), measured a higher activity against *S. aureus* than standard antibiotics like penicillin, methicillin, and ampicillin [135]. In another work, the biofilm eradication potential of different conventional disinfectants, namely DC&R^®^, VirkonS^®^, TH4++, Tek-Trol, and peracetic acid, alone and in combination with SNPs and CuNPs, was studied against *S. aureus* [136]. The biofilm eradication efficacy of the disinfectants loaded in SNPs and CuNPs reached 100% when the contact time and NPs’ concentrations increased. Based on these results, the authors claimed that drug-loaded NPs are the best choice to eradicate bacterial biofilms. Compared to gold and silver, copper NPs have lower production costs, and their therapeutic efficacy is beginning to be demonstrated in animal models (Table 3). Thus, CuNPs are believed to be the potential competitor of GNPs and SNPs for clinical translation [137].

#### 3.2.4. Metal Oxide NPs 

Other metals like zinc, titanium, iron, and magnesium have also found application in treating *S. aureus* infections [138]. Zinc is an essential trace element participating in several biological processes. The use of zinc oxide NPs (ZnONPs) was compatible with living cells and applicable in drug delivery and antimicrobial coatings. The previous concept of ROS induction for the antimicrobial activity of ZnONPs was questioned in the work by Kadiyala et al. [139]. In this study, the authors analyzed microarray results to study the antimicrobial mechanism and observed the upregulation of pyrimidine biosynthesis and carbohydrate degradation and down-regulation of amino acid synthesis and oxidative stress genes instead of induction of ROS generation. In another study by Liu and colleagues (2018), applying gelatine or ethylcellulose nanofibers containing ZnONPs was studied as active antimicrobial food packaging material against *S. aureus* [140]. The resulting nanofibers loaded with ZnONPs showed good surface hydrophobicity, water stability, and antimicrobial activity against *S. aureus*. It was also observed that the antimicrobial activity of ZnONPs increased from 43.7 to 62.5%, with simultaneous exposition to UV radiation. More recently, ZnONPs coated with the glycolipid surfactant rhamnolipid displayed promising results in an infection model (Table 3).

Iron oxide NPs were also tested to control staphylococcal infections. In a study of hyperthermia, Kim and coworkers found that streptavidin-functionalized magnetic iron oxide NPs had a two-fold increase in binding to protein A of *S. aureus* compared to IgG-conjugated NPs [141]. In another work, *S. aureus* causing osteomyelitis was controlled with Fe_3_O_4_ NPs implanted into the bone marrow cavity in mice (with hyperthermia) after methicillin-sensitive *S. aureus* (MSSA) infection [142].

Polyoxometalates containing tungsten (W), vanadium (V), molybdenum (Mo), or manganese (Mn) also display low minimum inhibitory concentrations (MIC), warranting further antibacterial studies [143]. Titanium oxide NPs (TiONPs) and magnesium oxide NPs (MgONPs) have been recognized as safe materials by the FDA [144,145]. These NPs are non-toxic, easy to obtain, and exhibit antibiofilm and antibacterial properties against Gram-positive and Gram-negative bacteria. Recently, the antibiofilm and antibacterial properties of TiONPs, MgONPs, and other metal oxide NPs, including those against *S. aureus*, were reviewed [144,146,147]. 

#### 3.2.5. Action Mechanism of Metallic NPs 

**Figure 3:**
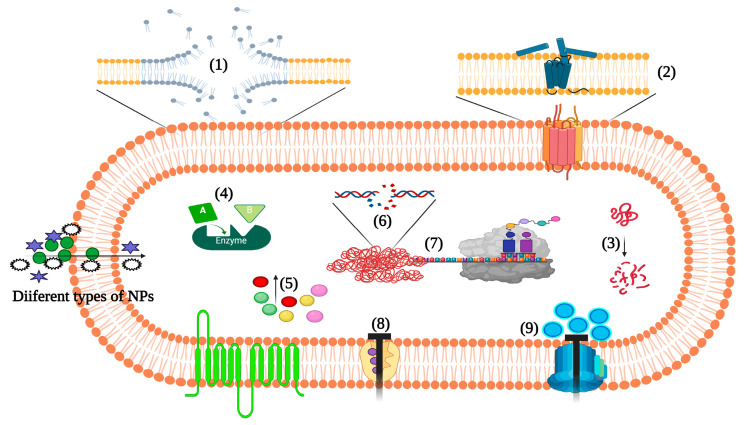
Schematic representation depicting the mechanisms of action of nanoparticles (NPs) with stronger experimental evidence. Metallic and other types of NPs can cause cell membrane disruption (1), membrane (2) and cytoplasmic (3) protein destabilization, inactivation of enzyme and metabolic functions (4), generation of reactive oxygen species (5), damage to DNA (6) and ribosomal (7) assemblies, and impairment of the transmembrane electron transport system (8) and efflux pumps (9).

The mechanism of bacterial growth and biofilm inhibition by NPs depends on several factors, such as surface area, surface interactions, NPs stability, NPs shape, and drug loading and releasing characteristics [148]. The interaction between NPs and the bacterial cell surface induces oxidative stress, enzymatic inhibition, and differential gene expression and protein function (Figure 3). The antibacterial actions of metal NPs are generally described as oxidative stress mechanisms, the release of metal ions, and non-oxidative mechanisms [149]. 

ROS-mediated oxidative stress is one of the most accepted mechanisms in the growth inhibition by NPs. ROS like superoxide (O_2_^−^) and hydrogen peroxide are generated by oxidative processes in the cells as natural byproducts, playing essential roles in cell survival, signaling, differentiation, and death. Aerobic metabolism leads to the production of ROS, which in turn are balanced by endogenous antioxidants, such as glutathione and other thiols, and enzymes like catalase or superoxide dismutase. However, upon additional insults leading to excessive production of ROS, oxidation of diverse biomolecules results in chronic cellular damage. Unbalanced ROS levels disrupt redox homeostasis, affecting the structure and functions of the bacterial cell membrane, proteins of different types, and DNA. Interestingly, the level of ROS production and the type of ROS generation seems to depend on the type of NPs used. Because of their increased surface-to-volume ratio, NPs can exhibit varied levels of antimicrobial efficacy via increased production of ROS and free radicals to tackle infections caused by MDR pathogens [149,150].

On release of metal ions by metal NPs, the cells can gradually take up the metal ions. Creeping into the intracellular compartments, the metal ions interact with proteins and nucleic acids via functional groups like amino, mercapto, and carboxyl groups [149], which alters the cell structure and interferes with the enzymatic and essential metabolic activity of the cells. Niemirowicz and co-workers in 2014 observed that the disulfide bond interaction between Au-superparamagnetic iron oxide NPs and key bacterial proteins hampered the bacterial metabolism and redox system [151]. Similarly, Su et al. in 2015 reported a significant alteration in the protein expression by CuONPs, thereby inhibiting the denitrification process [152]. Through proteomic analysis, the study revealed that CuONPs interfere with the functions of key proteins involved in nitrogen metabolism and electron transport. As pointed out before, NPs may also penetrate the bacterial cells through an absorption process, where the release of metal ions in the vicinity of the bacterial cells leads to the binding of metal ions to the negatively charged functional groups in the components of the bacterial cell membrane. For instance, protein coagulation happens when *S. aureus* cell membrane is adsorbed by SNPs [153]. 

Cell wall composition is crucial in the non-oxidative mechanism of NPs’ antimicrobial activity. Gram-negative bacteria possess a net negative charge on their cell surface because of components like phospholipids, lipoproteins, and lipopolysaccharides. In contrast, Gram-positive bacteria express only teichoic acids on the cell surface [154]. The presence of teichoic acids on the Gram-positive bacteria cell wall leads to the absorption of NPs [154]. Thus, it is expected that the antibacterial activity of NPs is stronger against Gram-positive than Gram-negative bacteria. 

Oxidative stress mechanisms are the best established in the anti-staphylococcal activity of NPs [112,129,134,139]. Wider investigations have already revealed alterations in different cellular components and pathways [129,134,139] that can contribute to important antibacterial effects like counteracting persister cells [124] and intracellular parasitism [133]. However, more research is needed to identify the key mechanisms of metal and other NPs’ action to regulate *S. aureus* virulence and pathogenicity.

#### 3.2.6. Silica-Based NPs

MSNs are an innovative strategy for the efficient delivery of drugs because of their biocompatibility and stability [155]. MSNs are not an antibacterial agent but can accommodate a huge payload of drugs and potentially deliver them to target tissues [155]. Gounani et al. studied the antibacterial properties of MSNs loaded with two different antibiotics, polymyxin B and vancomycin [156]. Using time-kill assays with different drug doses, the authors noticed that the antibiotic-loaded NPs were more effective than the free antibiotics, against *E. coli*, *P. aeruginosa*, and *S. aureus*. However, the results for minimum biofilm inhibitory concentration (MBIC) and minimum biofilm eradication concentration (MBEC) were found to be higher. Yet, the MSNs loaded with the antibiotics showed no toxicity to HEPG2, HEF-2, and HEK-293 cell lines. The authors hypothesized that MSNs did not enter the biofilm because of their size (>50 nm) or surface charge. 

A different approach investigated nitric oxide-releasing MSNs (NO-MSN) as *S. aureus* and *P. aeruginosa* biofilm eradicating agents [157]. The authors observed that NO-MSN activity was inversely proportional to their size. Comparing the shapes of NO-MSN, the rod-shaped ones were more active against preformed biofilms than the MSNs with a spherical shape. These findings were further validated by microscopic analysis, wherein the NO-MSNs with minimal sizes showed superior antibacterial actions. However, in the same study, NO-MSN at the MBIC and MBEC showed cytotoxic effects on fibroblast cells. 

To overcome the toxicity of NO-MSNs, other researchers used a combination of antibiotics and natural antimicrobials with MSNs, which may attenuate the adverse effects on non-targeted cells. For instance, Joyce et al. (2020) studied the antibacterial activity of rifampicin, a poorly water-soluble antibiotic, encapsulated in MSNs prepared by different methods (Table 3), against the small colony variant of *S. aureus* [158]. In a similar work, vancomycin administration via silicon NPs against MRSA-induced lung infection was studied in mice [159]. The resulting data indicated selective vancomycin delivery at the infection site causing a ten-fold reduction of the bacterial load compared to the free vancomycin treatment. In addition, the survival rate of mice was also stunning, close to 100% with the carried drug. Subsequently, other works proposed using drug-loaded MSNs incorporated in biopolymer films as active food packaging materials with prolonged drug-releasing capacity for more than 35 days [160,161]. Very recent studies from different authors have accentuated the therapeutic potential of antibiotic-loaded MSNs targeting bone MRSA infections (Table 3). 

An alternative strategy employing the coating of MSNs with natural cell membranes (for instance, erythrocytes, neutrophils, macrophages, or platelets) has gained interest in achieving target-specific drug delivery and avoiding the immune system [162,163]. Gentamycin-loaded MSNs with a fabricated lipid surface with peptide ubiquicidin on the surface was reported to target intracellular *S. aureus* and modulate inflammation-related gene expression [164]. Moreover, silica NPs containing deoxyribonuclease I and oxacillin eradicated *S. aureus* biofilm efficiently within 24 h [165]. In summary, different studies indicated that drug-loaded MSNs in diverse forms are a promising alternative to control *S. aureus* biofilms by way of slow drug release.

#### 3.2.7. Quantum Dots and Carbon Nanodots

Quantum dots (QDs) are semiconducting NPs exhibiting excellent size-dependent optical properties with high photostability. The more critical QDs are produced from carbon, metals (e.g., Si and Ge), metal chalcogenide, and metal oxides. Among the carbon-based QDs, carbon QDs, graphene QDs (GQDs), and graphene oxide QDs have often been studied for their bacteriostatic or bactericidal activities with photodynamic activity against several MDR pathogens under particular wavelengths. Usually, the size of the QDs varies between 2 and 10 nm. The size of the QDs determines the absorption and emission spectra of the particular QDs. The uses of QDs were reported in many fields like solar cells, fluorescent imaging, light-emitting diodes, and as antibacterial agents [15]. The QDs can easily be functionalized and provide additional advantages in targeted drug delivery. 

Curcumin (Cur) is a polyphenolic compound isolated from the plant *Curcuma*, which shows a wide range of biomedical applications, including in antimicrobial treatment. Still, the low bioavailability of Cur is a major disadvantage. Different nanoformulations improved Cur pharmacokinetics in animals and were well tolerated in human trials [105]. Singh et al. in 2017 formulated CurQDs in zirconia to enhance the stability and solubility of Cur [166]. Compared to the free Cur (175-350 µg/mL), CurQDs showed a lower MIC value (3.91-7.83 µg/mL). The formulated CurQDs at sub-MIC (0.0156 µg/mL) levels inhibited biofilm formation and eradicated preformed 3-day-old *S. aureus* biofilm. Based on microscopy analyses, the authors localized the CurQDs on the surface of the biofilm. Because of the particle size and the ability to interact with the preformed biofilm, CurQDs achieved a higher degree of biofilm penetration and cellular uptake. 

In some instances, controlling the stability, shape, and size of QDs during particle agglomeration becomes questionable. Nevertheless, these problems can be tackled by dispersing the QDs in a suitable matrix. Chitosan and cellulose are frequently used biopolymers as support matrices to improve QD applications [15,167], for example, cadmium selenide QDs. Wansapura and co-workers prepared a hybrid chitin film conjugating cadmium–tellurium QDs (CCT-QDs) via a facile aqueous synthesis route [168]. The preliminary studies with the CCT-QDs exhibited good antibacterial activity when placed over *S. aureus*- and *P. aeruginosa*-swabbed plates. Moreover, the material reduced biofilm formation by the pathogens and was evaluated by colony growth and CLSM studies. Likewise, GQDs were conjugated with acetophenone-substituted aromatic, macrocyclic, and organic compounds (phthalocyanine) by the self-assembly method. Later the self-assembled GQDs were allowed to form nanoconjugates with different metals via π- π interactions. Comparing the zinc and metal-free nanoconjugates, indium phthalocyanine showed high antibacterial activity with 9.68-log bacterial load reduction compared to zinc phthalocyanine with a 3.77-log reduction [169].

Fluorescent NPs termed CNDs with dimensions below 10 nm have been attributed with remarkable properties. CNDs are a new member of the NPs portfolio and were accidentally discovered in 2004 during the refining process of single-walled nanotubes [170]. These CNDs combine extraordinary features such as biocompatibility, easy functionalization, no inherent toxicity, water solubility, superior quantum yield, up-conversion photoluminescence, and others [171]. CNDs have been applied in various fields, including drug delivery and antibacterial studies [171]. Recent studies against *S. aureus* are listed in Table 3. Production of CNDs by green synthesis methods have received considerable attention because they are eco-friendly processes with low production cost [171]. In this regard, a very recent work by Lu and co-workers synthesized water-soluble CNDs from Cur and citric acid with potent antibacterial and antibiofilm properties against *S. aureus*, *B. subtilis*, *P. aeruginosa,* and *E. coli* [172].

**Table 3:** Recent studies on the control of *Staphylococcus aureus* growth and biofilm formation using different types of inorganic nanoparticles (NPs). The method of preparation (M), shape (S), average/particular size (AS), zeta potential (AZP), polydispersity index (PDI), encapsulation efficiency (EE), and drug loading capacity (DL) are indicated based on the original publication.

Reducing or Capping Agent/Encapsulated Drug	Properties of the NPs	Biological Activities	Reference(s)
**Gold NPs (GNPs)**			
*Padina tetrastromatica*-mediated synthesis of GNPs	**M**: Green synthesis **AS**: 1–20 nm **S**: Spherical **PDI**: ~23 nm	GNPs showed an MIC of 25 µg/mL Higher concentrations of GNPs also exhibited biofilm-eradicating ability	[173]
Polypeptide polymer-conjugated GNPs	**M**: Chemical reduction method **S**: Spherical **AS**: 23 nm **AZP**: 24 mV	Polypeptide-conjugated GNPs exhibited potent antibacterial activities against clinical isolates of MDR Gram-positive bacteria, such as MRSA Excellent in vitro and in vivo biocompatibility Studies with the antioxidant N-acetyl-L-cysteine suggested that oxidative stress is responsible for the antibacterial activity of these GNPs	[174]
Caffeine-loaded GNPs	**S**: Spherical **AS**: 77.9 nm	MIC was 512 µg/mL Biofilm inhibitory and biofilm eradication concentrations of 256 and 512 µg/mL, respectively GNPs eradicated persister cells of *S. aureus*	[124]
**Silver NPs (SNPs)**			
*Desertifilum* sp.-mediated synthesis of SNPs	**M**: Green synthesis **S**: Spherical **AS**: 4.5–26 nm	Comparing the growth inhibitory activity against different pathogens, MRSA was more susceptible to the SNPs (MIC 1.2 mg/mL) Anti-staphylococcal activity of SNPs was related to ROS-induced oxidative stress	[175]
SNPs	**M**: Microwave technique **S**: Spherical **AS**: 1-3 nm **AZP**: Positively charged	Interaction between SNPs and bacterial cell wall caused leakage of cytoplasmic material MIC of SNPs was 12.5 ppm against *S. aureus* Eradication of mixed species biofilms (*Candida albicans* and *S. aureus*) in a dose-dependent manner, with 0.53 ppm as the IC_50_ value SNP-functionalized catheter material was less prone to mixed species biofilm formation	[176,177]
Commercial SNPs	**AS**: 10 nm	Photolysis of staphyloxanthin via blue light increased the anti-staphylococcal activity of SNPs Blue light reduced the MIC of SNPs from 10 µg/mL to 1 µg/mL, which is safer for mammalian cells Photolysis of staphyloxanthin increased the uptake of SNPs into the bacterial cells	[178]
*Piper longum* mediated-synthesis of SNPs	**M**: Green synthesis **S**: Spherical **AS**: 10–40 nm	SNPs were active against *Bacillus cereus*, *S. aureus*, *Escherichia coli, Proteus mirabilis, Klebsiella pneumoniae, P. aeruginosa*, and *Salmonella typhi* SNPs were active after three months of storage	[179]
*Gardenia thailandica* leaf extract-mediated synthesis of SNPs	**M**: Green synthesis **S**: Spherical **AS**: 11.02–17.92 nm **AZP**: −6.54 ± 0.6mV	MIC of the SNPs against *S. aureus* ranged from 4 to 64 µg/mL SNP at 4 × MIC and 8 × MIC eradicated the *S. aureus* cells at 2 h and 1 h, respectively SNPs decreased the expression of efflux pump genes *norA*, *norB,* and *norC*	[180]
**Copper (CuNPs) and copper oxide NPs (CuONPs)**			
*Curcuma longa* or *Zingiber officinale* extract-mediated synthesis of CuNPs	**M**: Green synthesis **S**: Spherical **AS**: 20–100 nm	Agar well diffusion assay showed the antibacterial effect of CuNPs (1 and 5 mM) produced with *C. longa* was higher than those produced with *Z. officinale*	[135]
TH4+/CuNPs Virkon S/CuNPs Tek-Trol/CuNPs Peracetic/CuNPs DC&R/CuNPs	**AS**: The ranges of particle size were 79.88–100.62 nm (TH4+), 77.74–116.49 nm (Virkon S), 82.32–115.91nm (Tek-Trol), 90.25–105.07 nm (Peracetic), and 115.15–144.86 nm (DC&R) **AZP**: 2.92 and 3.43 mV	The ability of disinfectant-loaded CuNPs to eliminate the viable bacterial colonies in biofilm surfaces was studied with different concentrations and time points At a contact time of 5 min TH4+/CuNPs (1%), Tek-Trol/CuNPs (1%), DC&R/CuNPs (16%), 10 min Peracetic/CuNPs (0.5%), or 20 min Virkon S/CuNPs (2%), significantly reduced the total viable count of *S. aureus*	[136]
CuNPs and CuONPs	**M**: Plasma arc discharge method **AS**: 78 nm (CuNPs) and 67 nm (CuONPs)	Tested against different bacteria, CuNPs and CuONPs showed the highest zone of inhibition against *S. aureus* NPs induced ROS production, protein denaturation, DNA damage, and cell death	[112]
CuNPs	**AS**: 25 nm	CuNPs showed significant anti-staphylococcal activity with reduced toxicity against fibroblasts (at 6.25 µg/mL concentration) In vivo studies using *S. aureus*-induced mastitis rat model indicated that CuNPs improved clinical signs faster (three days) than gentamycin (four days) CuNPs reversed the *S. aureus*-induced histopathological changes in the mammary gland and, on the 5th day after treatment, bacterial load, mammary gland weight, and oxidative stress parameters were lower compared to the disease control and antibiotic-treated animals	[181]
**Other metallic NPs**			
*Lactobacillus plantarum* TA4-mediated synthesis of ZnONPs	**S**: Oval **AS**: 29.7 nm	ZnONPs were effective against *S. aureus* from poultry samples (disc diffusion assay) MIC and MBC values were 30 and 100 µg/mL, respectively ZnONPs inhibited biofilm formation in a dose-dependent manner The results suggested that ROS generation was the underlying antibacterial mechanism	[182]
Pancreatin-doped ZnONPs	**M**: Precipitation method **S**: Hexagonal **AS**: 85 nm	Antibacterial and virulence inhibitory activity against MRSA Protein leakage and generation of ROS were possible antibacterial mechanisms Pancreatin-doped ZnONPs sensitized the cells to vancomycin	[183]
Rhamnolipid-coated ZnONPs	**AS**: From 40 to 55 nm **S**: Spherical	NPs at 0.5 mg/mL had low toxicity to fibroblast cells and low hemolytic activity NPs treatment reduced the bacterial burden in infected wound in rats, revealing a rapid wound healing within five days compared to the rhamnolipid- and clindamycin-treated wounds In histopathological analysis, the NP-treated animals showed rapid remodeling of the epidermis and the presence of ample amounts of dermal cells on the 5th day of treatment	[184,185]
*Aspergillus terreus* S1 mediated-synthesis of MgONPs	**M**: Green synthesis **S**: Spherical **AS**: 8–38 nm **PDI**: 0.2	Growth inhibitory activity (MIC 200 μg/mL) against *B. subtilis* (13.3 ± 1.9 mm, inhibition zone), *E. coli* (11.3 ± 0.6 mm), *C. albicans* (12.8 ± 0.3 mm)*, P. aeruginosa* (14.7 ± 1.9 mm), and *S. aureus* (11.3 ± 0.6 mm)	[186]
*Carum copticum* extract-mediated synthesis of TiONPs	**M**: Green synthesis **S**: Spherical or spheroid shaped **AS**: ~12 nm	Inhibition of EPS secretion and rupture of preformed biofilms of *S. aureus*	[187]
*Ochradenus arabicus* leaf extract-mediated synthesis of TiONPs	**M**: Green synthesis **AS**: 26.48 nm	MIC of the TiONPs was 32 µg/mL TiONPs at 0.5 × MIC inhibited biofilm formation and EPS production by MRSA to approx. 50% MRSA strains increased production of ROS upon treatment with the TiONPs	[188]
**Mesoporous Silica NPs (MSNs)**			
Enzyme-functionalized MSN	**M**: Stober method **S**: Spherical **AS**: Lys@MSN (38 ± 5 nm), Ser@MSN (31 ± 7 nm), and DN@MSN (35 ± 4 nm) **AZP**: Lys@MSN (+12 ± 5 mV), Ser@MSN (−22 ± 5 mV), and DN@MSN (+27 ± 5 mV)	Enzymes lysostaphin (Lys@MSN), serrapeptase (Ser@MSN), and DNase I (DN@MSN) were immobilized in MSNs Lys@MSNs targeted MRSA and MSSA growth by inducing cell lysis The other two enzymes immobilized in MSNs targeted the biofilm formation of *S. aureus* by hampering the production of proteins and eDNA Lys@MSNs showed a 7.5- and 5-fold decrease in MIC and MBIC values compared to free lysostaphin	[189]
Rifampicin-loaded MSN	**M**: Solvent extraction (e) and calcination (c) methods **AS**: 40 nm (c & e), 80 nm (c) **AZP**: 15 (40e), 13 (40c), and 14 mV (80c) **DL**: 29 (40e), 33 (40c), and 38% (80c)	Hydrophilic e-MSN particles (prepared using solvent extraction) demonstrated a > 2-fold increase in Caco-2 cell uptake MSNs were efficacious against small colony variant *S. aureus* hosted within Caco-2 cells Compared to free rifampicin, the MSNs loaded with rifampicin reduced the level of *S. aureus* in Caco-2 cells 2.5-fold	[158]
Moxifloxacin/rifampicin-loaded MSN (gelatine/colistin coated)	**M**: Stober method **AS**: 396 nm **AZP**: -29.2 ± 0.65 mV	Antibiotic-loaded MSNs were studied against MRSA osteomyelitis both in vitro and in vivo MIC of the moxifloxacin and rifampicin MSNs were 3.906 and 0.977 µg/mL, respectively Intraosseous injection of MSNs decorated with aspartic acid hexapeptide (D6, affinity towards bone tissue) reduced *S. aureus* load to 92% in infected rabbit femurs within 24 h MSNs showed no toxicity towards osteoblasts and macrophages in vitro, but some effects on osteoclasts over time (72 h) NPs reduced biofilm formation and the expression of the proteases staphopain, SplF, and V8 protease, whereas they increased the expression of aureolysin and the transcriptional regulator protein Rot	[190]
Vancomycin-loaded MSN	**M**: Impregnation approach **S**: Spherical **AS**: 100 nm **AZP**: +26.5 mV	Antibiotic-loaded MSNs targeting bone and MRSA presented an MIC of 16 µg/mL Compared to treatment with free vancomycin, the targeted MSNs improved the recovery from orthopedic implant-related infections with MRSA in rats Hemolytic and studies with bone marrow mesenchymal stem cells indicated the biocompatibility of the MSNs, and no abnormalities were observed in the heart, spleen, liver, lung, or kidneys of treated rats	[191]
**Quantum dots (QDs) and Carbon nanodots (CND)**			
p-Coumaric acid QDs	**M**: Wet milling approach **AS**: 8.9 ± 3.7 nm **AZP**: −3.73 mV	Antimicrobial activity against a wide spectrum of foodborne microorganisms At minimal lethal concentration (250 µg/mL), 99.9% killing of bacterial cells was observed throughout the experiment time	[192]
Carbon QDs from gentamycin sulfate	**M**: Calcination method (180 °C optimal temperature) **S**: Spherical **AS**: 2–8 nm **AZP**: 10.9 mV	QDs effectively cleared bacterial pathogens like *E. coli* and *S. aureus* (MIC was 1.59 and 50.8 ng/mL at pH 5.5 and 7.4, respectively) QDs at 80 µg/mL eradicated (90%) preformed biofilms, whereas the gentamycin sulfate at the same concentration reduced only 10% of the biofilms QDs showed a low toxic profile against mammalian 3T3 cells, even at 2 mg/mL concentration	[193]
Carbon dots from m-aminophenol and tartaric acid	**M**: Hydrothermal method **S**: Spherical **AS**: 5–9 nm **AZP**: +33.2 ± 0.99 mV	The positively charged carbon dots showed anti-staphylococcal activity and low toxicity toward HeLa cells The carbon dots were selectively absorbed on the cell surface through electrostatic interactions	[194]
Carbon dots from levofloxacin hydrochloride	**S**: Spherical **AS**: 1.25 nm	MIC of the carbon dots against *S. aureus* was 128 µg/mL Mechanisms of electrostatic interaction for surface adherence and bacterial cell wall disruption were implicated in the antibacterial action No cytotoxicity was observed towards 293T cells (viability greater than 80% at a concentration of 100 μg/mL)	[195]
Negatively charged CNDs	**M**: Microwave-assisted synthesis **AS**: 2.5 nm **AZP**: −11.06 mV	Inhibitory activity against MRSA and vancomycin-intermediate *S. aureus* (MIC of 630 μg/mL)	[196]
CNDs from curcumin and citric acid	**M**: Hydrothermal method **AZD**: −15.1 mV	CNDs showed a broad range of antimicrobial and antibiofilm activity Bactericidal efficiency was maximal at 375 μg/mL against *S. aureus, E. coli, P. aeruginosa*, and *B. subtilis*	[172]

### 3.3. Organic NPs

Research on pharmaceutical development is exploring alternative ways to discover new molecules and to more efficiently deliver known drugs for improved treatments. In the same way as the inorganic NPs, numerous strategies have been developed for drug delivery and bacterial targeting through organic supports (Table 4).

#### 3.3.1. Lipid-Based NPs

Lipid-based NPs are efficient carriers to deliver drugs [197] and several lipidic nanosystems have been studied so far. Liposomes, solid lipid nanoparticles (SLNs), nanostructured lipid carriers, niosomes, quatsomes, micelles, nanodroplets (NDs), and nanoemulsions are among the lipid-based vehicles studied against MDR *S. aureus* [8]. 

In an exciting example, rifampicin was loaded into olein-derived NPs and investigated against MRSA [198]. The cryo-TEM study captured the nanoformulation based on cationic monoolein interacting with the MRSA cells. The MIC towards a MRSA strain was 0.025 μg/mL and, applied in vivo against skin infection in a mice model, improved the clinical symptoms, decreased *S. aureus* colonization, and modulated the immune response.

##### Liposomes

Liposomes are either nano- or micro-structured, closed spherical vesicles, generally composed of one or more layers of phospholipids. The constituent of phospholipids may be natural or synthetic, such as phosphatidylinositol, phosphatidylcholine (lecithin), phosphatidylserine, phosphatidylethanolamine, and phosphatidylglycerol [199]. In some instances, the use of cholesterol was reported to increase liposome stability and bilayer characteristics. Different categories have been used to distinguish the liposomes used in drug delivery, like conventional liposomes, pH-sensitive liposomes, immunoliposomes, cationic liposomes, and long-circulating liposomes. Yet, several studies pressed the evaluation of drug leakage and scale-up properties while developing liposomes, which may hinder the further progress of liposomes as drug carriers [199]. Liposomes are structural vesicles encapsulating the core bioactives to form nanoformulations, composed of either unilamellar or multilamellar patterns depending on the number of lipid bilayers. Effective liposomes protect from degradation and improve the pharmacokinetics and biodistribution of the therapeutic agents, while favoring their cellular uptake [200]. 

Antibiofilm efficiency and enhanced bacterial clearance ability have been reported with liposomes competent for successful drug release [201]. This is because the charge of the liposome confers their stability, promoting electrostatic repulsion, which aids their interaction with cell surfaces. This phenomenon may be the critical factor for the interaction of cationic liposomes towards the negatively charged cell wall of *S. aureus* [202]. This view has been substantiated by Dong et al. [203] that pointed that unilamellar liposomes of a cationic nature with minimal particle size inhibited *S. aureus* and their biofilms more efficiently than multilamellar vesicles. Although the biocompatible structure of liposomes holds both hydrophobic and hydrophilic bioactives within the vesicle to deliver into the site of infection [204], they require excipients for proper drug delivery [205]. To resolve this issue, surface functionalization/modification of liposomes has been proposed and established by binding a ligand to enhance liposomal stability. With this aim, Berti et al. (2016) examined calcium phosphate as a ligand and found that the calcium phosphate-coated liposomes discharged their load when in contact with *S. aureus* biofilm [206]. 

The increased stability of certain drugs after encapsulation is important for potential drugs having problematic unstable characteristics. Encapsulation of cinnamon oil showed an effective antibacterial and antibiofilm activity with higher stability [207]. However, Zomorodian et al. produced a nanoformulation by encapsulating silver NPs containing magnetic iron oxide within polyethylene glycol [208]. The activity of the prepared nanoformulation was comparable to that of the antibiotic tetracycline against *S. aureus*. These findings suggested that such NP encapsulation techniques may be appropriate to avoid certain drugs’ potential toxic side effects. Similarly, liposomal presentation of berberine and curcumin reduced the MIC values to concentrations that were achieved with other lipid-based carriers in pharmacokinetic studies (µg/mL levels in blood) [105]. Berberine is an alkaloid extracted from herbs, and its potential to suppress the QS system of MRSA was reinforced in a recent study of wound healing [209] and warrants further applications.

Another recent study assessed a liposomal system to eradicate *S. aureus* in vitro [210]. The most attractive part of the study was to reveal the outcome of three different liposomal nanoformulations of antibiotics, namely vancomycin, levofloxacin, and rifabutin against MSSA. Different lipid compositions of the liposomal formulation allow the antibiotics’ penetration and accumulation in the biofilm, thus favoring their therapeutic effect. Among the antibiotics, rifabutin showed a better antibiofilm activity and also significantly reduced the viable planktonic cells. In the same study, the liposomes presented no toxicity to osteoblast and fibroblast cell lines, especially the negatively charged formulations. Liposomes entrapping ciprofloxacin have also been employed in treating MRSA causing lung infections [211]. The nanosystem, based on PEGylated phosphatidylcholine, was taken up by macrophages in vitro, killing intracellular MRSA. After an intravenous injection into rats, the cargo was detected in the lungs and the MRSA infection could be reduced [211].

Targeted drug delivery has also been pursued in certain studies [159,212]. In this line, liposomes conjugated with mannose (ligand) have provided higher uptake into the biofilm matrix of *S. aureus* [213]. In a very recent work, Rani et al. showed the potential of targeted delivery of drug-loaded liposomes coated with a red blood cell membrane against MRSA [214]. The authors used a targeting ligand that enables the binding of the liposomes to the cell wall of *S. aureus* and the formulation reduced the MIC and evaded macrophage uptake. Biodistribution in rats indicated the presence of the liposomes in liver and blood, and a good safety profile was reported. 

Furthermore, a modified antimicrobial peptide loaded with antibiotics in liposomal vesicles has been examined in vivo (mouse model). Chol-suc-VQWRIRVAVIRK-NH2 (DP7-C)-modified azithromycin liposomes displayed an enhanced reduction in MRSA compared to both azithromycin in unmodified nanoformulation and the free drug [215]. 

##### Niosomes 

Niosomes are generally conceived as modified liposomes or non-ionic structured vesicles that consist of a hydrated mixture of cholesterol and non-ionic surfactants with high stability compared to liposomes [216]. Recent studies presented the use of niosomes in treating MDR *S. aureus*. For instance, Mirzaie et al. (2020) studied the effect of ciprofloxacin encapsulation in niosomes against ciprofloxacin-resistant *S. aureus* [217]. The MIC of ciprofloxacin was significantly increased against the 12 tested strains when loaded into niosomes (Table 4). More, sub-MIC levels of ciprofloxacin-loaded noisomes reduced biofilm formation of the *S. aureus* strains and the expression of the *icaB* gene. Likewise, another work studied the effect and drug delivery characteristics of two different niosomes loaded with ciprofloxacin against *S. aureus* [218]. The niosomes were prepared using the thin-film hydration method, with an entrapment efficiency of 77%. The antibacterial activity (MIC) of the efficient noisomal formulation was 8 to 32 times stronger against the tested isolates than the free antibiotic.

##### Quatsomes

Similar to niosomes, quatsomes are nanostructures inspired by liposomes developed for drug delivery purposes. Quatsomes are composed of cholesterol and cationic surfactants [219], are relatively stable for long periods, maintaining homogeneity in size, bilayer membrane organization, and lamellarity. 

A novel quaternary bicephalic surfactant was used along with cholesterol to synthesize pH-responsive quatsomes loaded with vancomycin to eradicate MRSA both in vitro and in vivo [220]. The prepared quatsomes were stable at room temperature and cold conditions for 90 days. Unloaded quatsomes showed pH-dependent activity against MRSA with an MIC of 125 µg/mL at acidic pH 6.0 and 250 µg/mL at neutral pH 7.4. Vancomycin loading substantially reduced the MIC values to 0.97 and 3.90 µg/mL at pH 6.0 and 7.4, respectively. The drug-loaded quatsomes showed no hemolytic activity at the test concentrations. Moreover, in vivo studies carried out using a BALB/c mice infection model revealed that vancomycin-loaded quatsomes significantly reduced the MRSA burden compared to the groups treated with unloaded quatsomes and free vancomycin. 

A recent work of Dong et al. demonstrated and compared the beneficial effect of cetylpyridinium chloride (CPC) quatsomes and CPC micelles against *S. aureus* and *P. aeruginosa* [221]. The CPC-micelles and CPC-quatsomes showed better activity against the planktonic cells of the tested microbes even at an exposure time of 5 min, with CPC quatsomes having higher activity. Further, the relative viability of biofilms and eradication of preformed biofilms were assessed using spectroscopic and microscopic analyses. The results revealed that the activity of micelles of quatsomes is purely time-dependent. In *S. aureus* biofilms, the relative biofilm killing activity was superior at 5 min of exposure time to CPC-micelles than the CPC-quatsomes. However, compared to CPC-micelles, enhanced activity was observed with CPC-quatsomes in eradicating the preformed biofilms of *S. aureus*. Further, exposure to the NPs for 10 min showed no toxicity towards human airway epithelial cells (NuLi-1). 

##### Micelles

Micelles are one of the excellent forms of NPs with good safety and used in clinics for drug delivery [222]. Micelles are amphiphilic molecules composed of an inner hydrophobic core and an outer hydrophilic face. The inner hydrophobic core is utilized to load and retain lipophilic drugs [223]. Coryxin, a novel lipopeptide and biosurfactant isolated from *Corynebacterium xerosis* NS5, showed potent antibacterial activity against *S. aureus*, *P. aeruginosa*, *E. coli*, and *Streptococcus mutans* [224]. By delivering coryxin in micelles, its efficacy was dramatically improved. The low critical micelle concentration of the biosurfactant was found to be 25 mg/L. The biosurfactant micelles showed anti-staphylococcal activity at a concentration of 0.19 mg/mL. In addition, these micelles could eradicate (83%) preformed biofilms of *S. aureus*. Against a Gram-negative organism, the activity of the coryxin micelles was less efficient. The differences in the formulation activity were correlated with the cell membrane composition of the Gram-positive and Gram-negative bacteria. 

Platensimysin, another antibiotic obtained from *Streptomyces platensis*, is active against MRSA. The clinical development of this drug has been hampered by its poor solubility and pharmacokinetic properties. In an exciting work, platensimysin was encapsulated in different micelles (Table 4) and assessed against extracellular and intracellular *S. aureus* and its biofilm formation [225]. The loaded micelles showed a profound activity against MRSA comparable to the free drug. Further, enhanced activity in macrophage-infected and peritonitis models and improved drug pharmacokinetics were observed for the platensimysin-loaded micelles. These works highlight the use of micelles as nanocarriers to increase the efficacy of drug candidates.

##### Stimulated Phase-Shift Acoustic Nanodroplets/Nanobubbles

Additional nano-structures have been investigated to inhibit *S. aureus* biofilm formation and its related infections, for example, stimulated phase-shift acoustic NDs to enhance biofilm eradication by antibiotics [226]. In this work, the phase shift NDs consisted of an inner liquid core composed of vancomycin encapsulated by perfluoropentane, and an outer lipid shell made of 1,2-dipalmitoylsn-glycero-3-phosphocholine and 1,2-distearoyl-snglycero-3-phosphoethanolamine-N-[methoxy(polyethylene glycol)-2000]. When exposed to an ultrasound or thermal energy threshold, NDs undergo a phase transition to a gaseous bubble. Later studies hypothesized that post-cavitation-generated gaseous bubbles might have mechanical alterations on tissues or cells. The NDs coupled with vancomycin were further subjected to low-intensity pulsed ultrasound and thermal energy thresholds to achieve vaporization and cavitation. The collective result of NDs and vancomycin was assessed against the preformed biofilms of *S. aureus*. The results revealed that the combined effect of ND and vancomycin rendered robust biofilm eradication and cell death compared to the free drug, which was confirmed via assessing the metabolic activity of the biofilm-residing cells and in vitro microscopy analysis [226]. 

Comparably, Argenziano and co-workers studied the drug delivery efficacy of nanobubbles (NBs) [227]. Here, the NBs were composed of vancomycin-loaded perfluoropentate as core material and an outer dextran sulfate shell coupled with the drug. Similar to NDs, NBs are also responsive to ultrasound and promote drug delivery. The ultrasound-dependent induction of antibiotic action was named the bioacoustic effect. In this work, the exposure of NB-coupled vancomycin to ultrasound enhanced the penetration of vancomycin, and the same was validated in vivo using a pig skin model. Based on the obtained data, the authors suggest the possible combined effect of ultrasound and NBs in treating *S. aureus* skin infections [227]. 

A recent work by Durham et al. [228] also delineated the potential of ultrasound-stimulated phase change contrast agents in aiding the penetration and eradication efficacy of antibiotics inside the *S. aureus* biofilms. Phase change contrast agents also consist of a liquid perfluorocarbon core and outer phospholipid shell as the stabilizing agent. Upon proper activation using ultrasound, the phase change contrast agent coupled with rhamnolipid and aminoglycoside antibiotics, namely mupirocin, vancomycin, linezolid, and rifampicin, significantly increased the activity of the antibiotics [228].

##### Solid-Lipid NPs 

Considering the importance of lipids in drug delivery, several different types of engineered NPs have been studied, such as SLNs and nanostructured lipid carriers. SLNs are nanocarriers composed of solid lipids and surfactants as stabilizers. SLNs have advantages over other carriers, namely high drug-loading capacity, targeted drug release, reduced side effects, controlled drug release, and increased stability, which favors their use in biofilm therapy [229]. Cefuroxime-loaded SLNs were synthesized and studied by Singh et al. [104] against *S. aureus* biofilm formation. The drug release assays indicated that the SLN released 54% of the drug in 2 h and 96% in 12 h. The SLN formulation resulted in 2-fold reduction of the MBIC of cefuroxime (40 µg/mL versus 80 µg/mL for the free drug). 

Apart from antibiotics, several other natural antimicrobial compounds have been loaded into SLNs to improve their stability, bioavailability, and antimicrobial activity. A work by Bazzaz et al. [230] studied the antibacterial activity of *Eugenia caryophyllata* essential oil loaded in SLNs against four different pathogens and highlighted its use for the successful delivery of plant essential oils in treating Staphylococcal infections. Similarly, Cur-loaded SLNs showed potential activity against biofilm formation by *S. aureus* [231]. Overall, these studies reinforce lipid NPs as effective drug delivery platforms with improved therapeutic potential for the treatment of *S. aureus* biofilms. Further studies with animal models are needed to support the therapeutic efficacy of lipid NPs against MDR bacterial infections, especially *S. aureus*.

#### 3.3.2. Polymer Based NPs

##### Polymeric NPs

NPs supported by biocompatible polymers can have high structural integrity and can thus be used to improve the stability of the loaded drugs and the NPs’ releasing properties. Above all, they are cost-effective, possess high storage potential, and can be easily synthesized [232].

The polymer poly(lactic-*co*-glycolic acid) (PLGA) is among the most studied for drug delivery, as PLGA presents good biocompatibility, so it is well accepted for clinical applications [105]. Several works reported using PLGA NPs to deliver antibiotics against bacterial biofilms and related infections. Hasan et al. (2019) studied the efficacy of clindamycin-loaded PLGA NPs (CPN) and clindamycin-loaded PLGA-polyethylenimine NPs (CPPN) against MRSA in a wound infection model [233]. Compared to the negatively charged CPNs, the positively charged CPPNs showed an enhanced anti-MRSA activity with a 5-log reduction (99.999% killing) at a concentration of 0.5 mg/mL. Another recent comparison of positively (poly-lysin-coated) and negatively charged (poly-lactic acid) NPs is presented in Table 4. With the CPPNs, wound healing was measured as 94%, whereas with the CPN, it was 64% on the 8th day post-injury. Finally, the data indicated that positively charged CPPNs effectively heal wounds by reducing the *S. aureus* burden at the infection site. Likewise, Thomas et al. [234] studied the efficacy of ciprofloxacin-loaded PLGA NPs against biofilm formation by *S. aureus*. The study revealed that drug release by the prepared NPs was 50–60% within 24 h, and complete release was observed on the 5th day. Further, the minimum biofilm eradication concentration of the ciprofloxacin-loaded PLGA NPs was determined against 5-day-old *S. aureus* biofilms. After 24 h of incubation, the minimum biofilm eradication concentration was found to be 128 and >256 µg/mL for free and encapsulated ciprofloxacin, respectively. It was speculated that incomplete drug release by the PLGA NPs at 24 h might be the reason for the different efficiency. 

Chitosan is another well-studied polymer as a drug carrier, owing to its beneficial advantages like being low in cost, biodegradable, and biocompatible. Unlike PLGA, chitosan is a natural polymer that is often used to encapsulate various drugs, including inorganic NPs, natural compounds, and essential oils [105,235,236,237,238]. Given the prominence of *S. aureus* infections in dairy farms, chitosan NPs and drug-loaded chitosan NPs have been studied to contain *S. aureus*, in intracellular infections and associated with intramammary infections and mastitis [239,240]. Asli et al. [239] investigated different forms of chitosan and underlined the antibacterial, antibiofilm, and safety profile of the 2.6 kDa form. Breser et al. [240] also measured the effect of the NPs on the secretion of cytokines by bovine mammary epithelial cells and noted changes in the levels of IL-6. Chitosan coupled with cefotaxime showed anti-MRSA and antibiofilm activity as an efficient biocompatible nanocarrier [241]. Owing to their mucoadhesive property, Silva and co-workers used chitosan NPs encapsulating daptomycin to treat ocular infections [212]. The daptomycin-loaded chitosan NPs required 4 h to achieve high drug delivery and anti-MRSA activity. Loading the flavone chrysin in chitosan NPs enhanced its anti-biofilm efficacy [238]. In other words, chitosan nanoformulations were effective against *S. aureus* biofilm formation and drug-resistant strains (Table 4).

Alginate is another polysaccharide offering low toxicity and easy manipulation and is already used for wound healing [242]. Alginate/chitosan NPs were tested with rifampicin encapsulation against MSSA and MRSA in pulmonary intracellular infections [243]. The results revealed that the alginate NPs have great potential as novel antimicrobial nanomaterials [242,244].

##### Dendrimers

These are multi-branched polymers surrounded by layers around the core unit [245]. They can accommodate high-density hydrophilic and hydrophobic drugs and functional groups can be added to these nano-structures for targeted delivery [245,246]. In this context, platensimycin, known to target proteins responsible for fatty acid biosynthesis, was encapsulated in two different forms of NPs, namely poly(amidoamine) (PAMAM) dendrimers and PLGA NPs [247]. Compared to the free platensimycin, the platensimycin-loaded NPs showed enhanced pharmacokinetics and higher activity against MRSA biofilms in vitro at concentrations below μg/mL. Some intrinsic anti-biofilm activity of (unloaded) PAMAM and PLGA NPs was detected only at high concentrations. Studies with different models indicated that the loaded NPs function as drug carriers through Caco-2 cell monolayers, can be internalized by macrophages, and are effective against MRSA infections (Table 4). A previous work studied the antibacterial activity of PAMAM against *P. aeruginosa*, *E. coli*, *Acinetobacter baumanni*, *Shigella dysenteriae*, *Klebsiella pneumonia*, *Proteus mirabilis*, *S. aureus*, and *Bacillus subtilis* [248]. The synthesized PAMAM showed antibacterial activity against all the tested bacteria, and relatively low cytotoxicity on HCT 116 and NIH 3 T3 cells. The action of PAMAM was assessed by disc diffusion and broth dilution methods, and the MIC against *S. aureus* was in the μg/mL range.

Conjugation of two NPs is expected to increase the activity while lowering the effective concentration of the individual NPs. To test this hypothesis, a recent work assessed the antibacterial activity of PAMAM dendrimers with dual-conjugated vancomycin and SNPs against VRSA [249]. These heterofunctionalized dendrimers afforded very relevant anti-VRSA properties in vitro and rapid wound healing actions (Table 4). In the same line, other works confirmed the potential of dendrimers conjugated with antibiotics for the treatment of *S. aureus* infections [250,251]. The encapsulation of vancomycin into hybrid dendrimer–polymeric vesicles enabled a slow release of the drug for 48 h, and a more effective anti-MRSA outcome compared to the free drug, but the intrinsic activity of the vesicles was not assessed in this work [251].

Nevertheless, dendrimers with potent intrinsic anti-MRSA activity were also reported. Without any drug loaded, organometallic dendrimers [252] and a lipidated peptide dendrimer [253] presented MICs towards MRSA in the low micromolar or μg/mL range. Moreover, a new type of polyurea dendrimers mimicking antimicrobial peptides was recently introduced as an alternative to PAMAM dendrimers [254]. The best formulation yielded very promising results against several microorganisms, including MRSA in vivo (Table 4). A common mechanism of these various dendrimers is to cause membrane disruption leading to rapid cell death. 

##### Cyclodextrins 

Cyclodextrins (CDs) are cyclic oligosaccharides with glucopyranose units as the monomer. CDs generally have inner lipophilic and outer hydrophilic moieties capable of accommodating a variety of large molecules through non-covalent inclusion complexes [255]. 

Several studies utilize CDs as a capping agent to enhance the antibacterial efficiency of the source compounds [256]. In addition, CDs are known to ameliorate the irritations caused by drugs [257]. It was reported that β-CDs could enhance artemisinin’s solubility and antibacterial activity against MRSA [258]. Different CDs loaded with the polyphenol compound caffeic acid were also tested against *S. aureus* [259]. In the same line, the inclusion complex of the phytochemical monocyclic sesquiterpene α-bisabolol was assessed for its antibacterial activity against *S. aureus*. α-bisabolol/β-CDs alone and in combination with gentamycin showed strong activity against MDR *S. aureus* [260]. Hence, in general, it is believed that encapsulation of the drugs into CDs increases their efficacy with low toxicity concerns.

**Table 4:** Recent studies on controlling *Staphylococcus aureus* growth and biofilm formation using different types of organic nanoparticles (NPs). The method of preparation (M), shape (S), average/particular size (AS), zeta potential (AZP), polydispersity index (PDI), encapsulation efficiency (EE), and drug loading capacity (DL) are indicated based on the original publication.

Reducing or Capping Agent/ Encapsulated Drug	Properties of the NPs	Biological Activities	Reference
**Liposomes**			
Lecithin and Tween-80 liposomes with *Laurus nobilis* leaf extract	**M**: Ultrasound **AS**: 99.05 ± 2.98 nm **EE**: 73.76 ± 1.10%	MIC and MBC of plant extracts were between 100 and 500 ppm At 1500 ppm, the loaded liposomes inhibited oxidation, bacterial growth, and spoilage of minced beef inoculated with *E. coli* and *S. aureus*	[261]
Lecithin liposomes with co-encapsulated berberine and curcumin	**M**: Film hydration **AS**: 253 ± 22 nm **AZP**: −57 ± 4 mV **EE**: 57 ± 3%	MIC of free berberine and curcumin were 62 and 250 µg/mL, respectively Encapsulation reduced the MIC of the drugs by approximately half and more efficiently prevented MRSA biofilm formation Free berberine and curcumin combinations showed an MIC of 31/16 µg/mL with an FIC index of 0.56 (no interaction), while the dual drug-loaded liposomes showed an MIC of 8/10 µg/mL with an FIC index of 0.13 (synergy) The liposomes were more efficient than clindamycin in reducing intracellular infection	[262]
**Niosomes**			
Ciprofloxacin-loaded niosomes	**M**: Remote-loading technique **S**: Spherical **AS**: 123 nm **PDI**: 0.198 **EE**: 79.25%	Stable ciprofloxacin-loaded niosomes showed MIC in the range of 2–4 µg/mL against the *S. aureus* strains, a 4- to 5-fold increase in antibacterial potency compared to the free drug Sub-MIC inhibited the biofilm formation of ciprofloxacin-resistant *S. aureus* and down-regulated the *icaB* gene	[217]
Cefazolin-loaded niosomes	**M**: Film hydration **S**: Spherical **AS**: 100 nm **AZP**: −63 mV	Cefazolin-containing niosomes removed one- to five-day-old biofilms in a concentration-dependent manner (MRSA isolates from patients with pressure sores and diabetic ulcers) Histopathological results indicated that mice treated with cefazolin-loaded niosomes recovered faster than those treated with the free drug or the untreated group	[263]
**Quatsomes**			
Vancomycin-loaded quatsomes from quaternary bicephalic surfactants and cholesterol	**M**: Sonication/dispersion method **AS**: 123 nm **AZP**: 0.169 mV **EE**: 52.2%	The pH-responsive quatsomes showed 32- and 8-fold lower MICs against MRSA at pH 6 and 7.4, respectively, compared to the free vancomycin The drug-loaded quatsomes caused more significant membrane damage, had a bactericidal effect, and counteracted MRSA biofilms in vitro In a mouse skin infection model, the quatsome formulation performed better than the free antibiotic	[220]
Cetylpyridinium chloride (CPC)-quatsomes	ND	No toxicity towards human airway epithelial (NuLi-1) cells Low concentration inhibited the planktonic and biofilm cells of *S. aureus* and *P. aeruginosa*	[221]
**Micelles**			
Platensimycin-loaded micelles constructed using [poly(lactic-co-glycolic acid)-poly(2-ethyl-2-oxazoline) (PLGA−PEOz)] and PLGA-poly(ethylene glycol) (PLGA-PEG)	**AS**: 183 nm (PLGA−PEOz), 195 nm (PLGA-PEG) **AZP**: -5.37 (PLGA−PEOz), −5.42 (PLGA-PEG) **EE**: 41.7% (PLGA−PEOz), 40.4% (PLGA-PEG)	Improved results against intracellular MRSA in a macrophage infection model Compared to the free drug, drug-loaded micelles showed higher potential against MRSA-induced peritonitis in mice (dose 20 mg/kg, increased survival and reduced colonization) The drug-loaded micelles were not toxic to the cells nor the animals Cmax after i.p. injection of the free drug was 28 ± 9 μg/mL, but concentrations greater than 50 μg/mL were measured after administering the encapsulated drug	[225]
**Solid Lipid NPs (SLNs)**			
Curcumin-loaded SLNs	**M**: Microemulsion method **S**: Spherical **AS**: 126.87 ± 0.94 nm **PDI**: 0.21 **ZP**: 30 ± 0.3 mV **EE**: 99.96% **DL**: 1.8%	Curcumin SLNs were effective against pathogens such as *S. aureus* and *E. coli* Lower MIC value (142 μg/mL) than free curcumin (1000 μg/mL) The curcumin SLNs reduced the pathogens’ cell counts in contaminated food for eight days	[264]
Anacardic acid encapsulated in SLNs	**M**: Hot homogenization **S**: Spherical **AS**: 203.6 ± 3.05 nm **PDI**: 0.277 ± 0.02 **ZP**: −21.4 ± 2.81 mV **DL**: 76.4 ± 1.9%	Stable for 90 days and non-toxic to the human keratinocyte cell line HaCat High anti-staphylococcal and biofilm inhibitory activities	[229]
**Polymeric NPs**			
Rifampicin-loaded poly-lactic acid NPs	**M**: Nanoprecipitation **S**: Spherical **AS**: 144 nm **PDI**: 0.08 **AZP**: −56 ± 5 mV **DL**: 2.2% **EE**: 90.5%	NPs coated with poly-lysine were more active against the growth and biofilms of *S. aureus*, presumably due to enhanced interaction and slow penetration into *S. aureus* biofilms	[265]
*Citrus reticulata* essential oil loaded in chitosan NPs	**AS**: 131–162 nm **EE**: 67.32%–82.35% **AZP**: 30 mV	The loaded NPs disturbed bacterial cell membranes and displayed high anti-staphylococcal activity, as well as inhibition of biofilm formation and premature biofilms of *S. aureus*	[266]
Chitosan functionalized SNP by *Sygyzium aromaticum*	**M**: Biogenic synthesis **S**: Spherical **AS**: 30–40 nm	Effective against MRSA and VRSA Lethal toxicity towards HeLa cells and brine shrimp was observed at 325 μg/mL, which is three times higher than the effective concentration showing anticoagulation, antiplatelet, and thrombolytic activities	[267]
**Dendrimers**			
Platensimycin-loaded PLGA and PAMAM dendrimer NPs	**M**: Emulsification-evaporation **AS**: 175.6 nm (PLGA) and 218.1 nm (PAMAM) **PDI**: 0.10 (PLGA) and 0.17 (PAMAM) **AZP**: −17.7 mV (PLGA) and 17.2 mV (PAMAM) **DL**: 7.81% (PLGA) and 8.42% (PAMAM) **EE**: 62.1% (PLGA) and 63.2% (PAMAM)	Inhibited MRSA growth and biofilms and killed the bacteria in a macrophage cell model more efficiently than the free drug Treatment with both types of drug-loaded NPs was effective against MRSA peritoneal infection in the mice models, with reduction of MRSA in the blood and kidneys, and full survival for 7 days, while the animals treated with the same dose of free drug (10 mg/kg, i.p.) died in 24 h In pharmacokinetic study in rats, the NPs formulations provided a 2- to 4-fold higher AUC and extended the mean residence time of the drug (Cmax approx. 80 μg/mL) Loaded PLGA and PAMAM NPs showed no appreciable effect on RAW 264.7 cell viability at concentrations well above those providing antibacterial activity (below 100 μg/mL)	[247]
PAMAM dendrimers with amide-conjugated vancomycin and incorporated SNP	**M**: Drug-PAMAM with amide conjugation **AS**: Dual drug-conjugated dendrimers with 68 nm **AZP**: 27.5 mV	5–7-log reduction in colony-forming units of VRSA Antimicrobial resistance induction was not detected in a susceptible strain, in contrast to using the free antibiotic Good biocompatibility with IH 3T3 fibroblasts and HUVEC cells (up to 8 µg SNP/mL) and low hemolytic effects Irrigation of infected wounds in mice with the dual-drug dendrimers cleared VRSA and reduced the accumulation of granulocytes at the wound site more efficiently than the free antibiotic or the SNP-only PAMAM dendrimers	[249]
Polyurea (PURE) oligoethyleneimine (OEI) dendrimers	**M**: Grafting oligo-(2-ethyl-oxazoline) in polyurea dendrimer, followed by acid hydrolysis **AZP**: cationic Mw 82,871 g/mol (PURE-G4-OEI-48) and 160,788 g/mol (PURE-G3-OEI-24)	MIC and MBC against MRSA, MSSA, *Streptococcus pneumonia*, Gram-negative bacteria and *Candida* strains below 10 μM (lower than 1 μM in the case of MRSA) PURE-G4-OEI-48 effective against *Pseudomonas aeruginosa* and MRSA infections in a *Galleria mellonella* insect model Up to 6 μM, no toxicity was observed against human bronchial epithelial 16HBE14o- and vaginal VK2 (E6/E7) cell lines, nor an effect on the health index scores of *G. mellonella* Live/dead assays, SEM, and molecular dynamic simulations supported a fast-killing mechanism via membrane disruption	[254]

## 4. Application of NPs in Antimicrobial and Antibiofilm Coatings

In medical and surgical settings, biofilm formation by *S. aureus* is of particular concern because of its ability to cause device-related infections, delays in the treatment process, implant failure, and recurrent infections and surgeries [3]. Device-related infections, other pathologies, and pathogens are risk factors for mortality [268]. 

Several strategies have emerged to tackle this issue, such as the introduction of anti-adhesive and bacterial-repellent surfaces, reviewed previously [41,269]. Bacterial adhesion is modulated by the physical and chemical properties of the surface, namely by the presence of specific functional groups, surface hydrophobicity, and net electric charge [269]. Bacteria-repelling surfaces are usually inert, and the repellent activity can be related to a self-assembled monolayer, hydrogel coating, or other methods that modify surface morphology or topology. On the other hand, coating materials with intrinsic antibacterial and antibiofilm characteristics may be used to provide bacteria-repelling activity to the surfaces of devices [269]. An example is a functionalization with NPs bearing antibiofilm and antibacterial properties [8,270]. 

Several works have investigated the use of NPs fabricated onto the surfaces of titanium, catheters, poly (ether ketone) (PEEK), and other materials. Implants with titanium surfaces are widely applied in dentistry and orthopedic treatments. In one study, SNPs anchored to titanium implants showed a steady release of Ag^+^ ions for 28 days [271]. SNP-coated titanium plates showed antibacterial activity against *S. aureus* through a unique trap-killing mechanism. Using SNP-anchored titanium avoided abnormal osteoblast activities and host inflammatory responses. Similarly, the application of NPs in different implant surfaces such as silk fiber, stainless steel, metal cotton, and hydrogels has been reviewed [272]. 

Catheters are used in drug administration, nutritional support, and other fluid administration through the venous channel. Likely, the infected catheter directs the MDR pathogens to veins and arteries. Hence, the infections in catheters are the origin of many hospital-acquired diseases, for which the antibacterial coatings of catheters are of great interest. With this in mind, chlorohexidine-loaded nanospheres were assessed in silicon urinary devices [273]. The drug release lasted up to 15 days and had a beneficial effect on MDR pathogens causing urinary tract infections such as *S. aureus*, *S. epidermidis,* and *P. aeruginosa*. A recent study by Srisang et al. (2020) assessed the multilayer coating of chlorohexidine-loaded NPs (micelles and nanospheres) in Foley urinary catheters [274]. The coated catheter showed good activity against *E. coli* and *S. aureus*, prolonged drug release up to 28 days, and no toxicity to fibroblast L929 cells. Likewise, the study by Sehmi and co-workers delineated the use of CuNPs embedded in silicon or polyurethane polymers, as these are the preferred material for catheter synthesis [275]. The catheter materials with encapsulated CuNPs showed potent activity against *S. aureus*, with the polyurethane-CuNPs material achieving 99.9% bacterial reduction within two h. An innovation was introduced, having in mind ventilator-associated pneumonia, by combining the potentials of the phytochemical curcumin and photodynamic therapy [276]. Conjugation of the polyphenol to the endotracheal tube decreased bacterial adhesion and biofilm formation. Further, the polyphenol conjugation enabled a robust photodynamic inactivation (under blue light at 450 nm) of *E. coli*, *P. aeruginosa*, and *S. aureus*.

Medical implants like heart valves and dental and bone implants are fabricated from PEEK. Coating PEEK/poly (ether imide) with titanium oxide NPs showed antibacterial activity against pathogens like *E. coli* and *S. aureus* [277]. Similarly, Chen and his colleagues constructed a PEEK implant with vancomycin conjugated to gelatine NPs [278]. This new composite material gradually released vancomycin, showed no toxic effects, and had good interaction with osteoblast cells. 

The cochlear implant is a gold standard treatment option for aiding hearing loss, but they are susceptible to biofilm formation by pathogenic bacteria. Co-deposition of ZnO with MgF_2_ in the cochlear implant was found to reduce biofilm formation by clinically relevant pathogens such as *S. aureus* and *Streptococcus pneumoniae* [279]. This combination significantly inhibited bacterial growth when compared to the activity of individual NPs. Moreover, fibroblast cells from the human auditory canal were used to assess the cytotoxicity of the tested combination, and the result was satisfactory. 

Besides medical implants, medical textiles such as hospital bed sheets, bandages, and uniforms should possess antibacterial properties, which could reduce the incidence of hospital-acquired infections [280]. Interestingly, several works studied the potential use of hospital textiles coated (sonochemically) with ZnONPs against medically relevant pathogens such as *S. aureus* and *E. coli* [281,282]. Use of the ZnONPs-coated fabrics by patients in the form of hospital textiles, such as pillow covers, bedspreads, and patient gowns, significantly reduced hospital-acquired infections [281]. Thus, incorporating NPs in the fabrication processes provides a promising alternative or complementary strategy to contain hospital-associated infections.

## 5. Challenges and Prospects of Research in the Field

### 5.1. Biological Behavior of NPs

As discussed earlier, NP treatment holds promising potential for treating different human diseases, not only bacterial infections. Besides their medical applications, many important questions remain unsolved for NPs’ transition from the lab to the market. The significant uncertainties to address in the clinic application of NPs are related to their bioavailability, safety, and compatibility [283]. The knowledge related to long-term exposure to NPs is still at large. However, it is well known that physiochemical properties like the shape and surface chemistry alter NPs’ interactions with biological systems [284,285,286], and their pharmacokinetics properties (absorption, distribution, metabolism, and excretion), which will all have a role on the final biological effects [287]. 

Most of the research studies assess the biocompatibility and acute toxicity of NPs using cell lines in vitro [288,289]. Nevertheless, the administration of therapeutic NPs includes dermal application, inhalation, ingestion, or intravenous injection. Therefore, it is vital to assess the biocompatibility of NPs in higher animal models. Several reports point to the adverse effects of NPs when administered through different routes. For instance, toxicity was observed in vital organs like the lungs, spleen, liver, and heart when NPs were administrated by inhalation [290]. Similarly, upon intravenous injection, the accumulation of NPs was observed in the colon, lung, bone marrow, and liver. However, these reports provided limited information on the interaction between the specific cells and the NPs or on the mechanisms through which NPs induce adverse effects. These clues are crucial for future research aiming to develop safer nanotherapeutics through surface modifications [8,284]. In this light, European and US initiatives have issued some relevant statements to address the apprehensions about the biosafety of nanomaterials [287]. However, to date, there are no specific regulations to assess the biocompatibility, metabolism, and clearance of NPs [291].

### 5.2. Limitation in NPs Production

Production technologies are yet another pit stop in shifting the NPs from the lab to the market. The development of novel production methods is vital for the large-scale production of NPs at affordable costs. Although the cost of nanosystem production is very high, the financial outcome cannot be predicted in the early stages of production. 

There are several nanotherapeutics in the clinical market, for instance, liposomes carrying amphotericin B (Ambisome^®^), ritonavir (Norvir^®^), and morphine (DepoDur^®^) [292,293,294]. Nevertheless, as per the published reports, the US Food and Drug Administration and European Medicines Agency have approved the use of nearly 70 nanomedicines against human diseases since 1995, while twice the number of formulations are now either at an early stage or finishing stage of clinical studies [295]. In general, these reports from the US Food and Drug Administration and the European Medicines Agency show that numerous studies have been done to overcome the pitfalls and exploit nanosystems’ potential in medical applications.

## 6. Conclusions and Future Outlook

The emergence of MDR strains poses serious concerns to human health on a global scale. The growing number of infections and MDR *S. aureus* cases, the inevitable health and economic losses, and the lack of effective treatments to control *S. aureus* resistance mechanisms have boosted the investigation of novel therapeutic strategies. To this end, some nanosystems discussed in this ample review were used as carriers to enhance the bioavailability and efficacy of antimicrobials by improving the drug targeting, release profile, and adverse effects. Different forms of NPs with antibacterial or antibiofilm activities have also been discussed herein. Thus, it is clear that the studies are beginning to scratch how the interaction between NPs and the bacterial cell surface induces oxidative stress and affects enzymatic activity, gene expression, and protein function. 

Although the underlying molecular mechanism is unknown, the success of drug combinations against MRSA [296] indicates that drugs rendered ineffective by the development of antibiotic resistance can still be valuable. Indeed, evidence has accumulated on the synergistic combination of NPs with conventional drugs against *S. aureus* infections. However, the studies are only starting to uncover the effect of these combinations on persister cells [124] and on specific proteases [190]. 

As discussed above, nanoformulations were found to successfully target the initial steps of bacterial adhesion in biofilm formation by reducing the cell surface hydrophobicity and exopolysaccharide production [238,297]. More importantly, some NPs inhibit the transcription of biofilm-related genes like *icaA*, *icaB,* and *icaD* [217,298] or efflux pump genes [180]. Nevertheless, it is evident there is a gap in understanding the influence of NPs on key virulence factors (Table 1 and Table 2). The modulation of TCS regulating biofilm formation, stress response, or antibiotic resistance warrants further investigation, as well as the understudied effects on the quorum sensing system and RNAIII-mediated production of toxins and other factors (Figure 2). In Gram-negative bacteria, a dendrimer was described to bind to and antagonize lipopolysaccharide activity [299], suggesting that NPs could be designed to block key cell surface components like adhesins in *S. aureus*. 

In many cases, the biological impact of NPs has been examined in vitro, and little is known about their potential activity in animal models. However, some nanoformulations have already returned encouraging results in animal models, namely, SNPs [298], dendrimers [247,254], and antibiotic-loaded lipid NPs [198] and MSNs [190,191,300]. Nevertheless, a more comprehensive evaluation of critical aspects such as toxicokinetics, translocation, and synchronized response of different tissues to NPs needs further research. 

Concurrently, the advances in nanofabrication and biomanufacturing technologies can also be expected to contribute to realizing the promise of safe and efficient nanotherapeutics. Keeping the pros and cons of NPs in mind, future efforts in the standardized production and the clinical evaluation of nanoformulations will promote nanotechnology-based treatments of MDR pathogens such as *S. aureus*.

## Figures and Tables

**Table 1:** Critical virulence processes and associated genes in *Staphylococcus aureus* pathogenesis [23].

Virulence Processes	Virulence Factors Involved	Responsible Genes
Attachment	Microbial surface components recognizing adhesive matrix molecules involving surface proteins such as bone sialoprotein-binding protein, fibronectin-binding proteins, clumping factors, and fibrinogen-binding protein	*bbp*, *fnbA*, *fnbB*, *clfA*, *clfB*, *sdrD*, *sasG*, *fib*, and *cna*
Invasion or tissue penetration	Enzymes able to break down lipids, phospholipids, proteins (elastin), DNA, and hyaluronic acid	*hysA*, *nuc*, *gehB*, *plc*, *sepA*, *sspA*, and V8
Destroying or evading host immune system	Pore-forming toxins like leukocidins, phenol-soluble modulins, protein A, CHIPS, Eap, staphyloxanthin, staphylococcal complement inhibitor, and capsular polysaccharides	*lukS*-PV, *hlg*, *lukF*-PV, *crtN*, *spa*, *psm*-a gene cluster, *chp*, *scn*, *eap*, cap5, and 8 gene clusters
Persistence and tolerance	Factors involved in intracellular persistence and biofilm formation such as polysaccharide intracellular adhesions, and formation of small colony variants	*ica* operon, *dnaK*, and *hemB* mutation
Toxins mediated infections and sepsis	Toxic shock syndrome toxin-1, α-toxin, enterotoxins, exfoliative toxins A and B, and lipoteichoic acid	*sea*, *hla*, *tstH*, *eta*, and *etb*
